# Intricate Distribution Patterns of Six Cytotypes of *Allium oleraceum* at a Continental Scale: Niche Expansion and Innovation Followed by Niche Contraction With Increasing Ploidy Level

**DOI:** 10.3389/fpls.2020.591137

**Published:** 2020-12-09

**Authors:** Martin Duchoslav, Michaela Jandová, Lucie Kobrlová, Lenka Šafářová, Jan Brus, Kateřina Vojtěchová

**Affiliations:** ^1^Plant Biosystematics and Ecology RG, Department of Botany, Faculty of Science, Palacký University, Olomouc, Czechia; ^2^Institute of Botany, Czech Academy of Sciences, Pruhonice, Czechia; ^3^Department of Geoinformatics, Faculty of Science, Palacký University, Olomouc, Czechia

**Keywords:** cytogeography, chromosome numbers, ecological niche, flow cytometry, geophytes, ploidy coexistence, polyploidy

## Abstract

The establishment and success of polyploids are thought to often be facilitated by ecological niche differentiation from diploids. Unfortunately, most studies compared diploids and polyploids, ignoring variation in ploidy level in polyploids. To fill this gap, we performed a large-scale study of 11,163 samples from 1,283 populations of the polyploid perennial geophyte *Allium oleraceum* with reported mixed-ploidy populations, revealed distribution ranges of cytotypes, assessed their niches and explored the pattern of niche change with increasing ploidy level. Altogether, six ploidy levels (3*x*−8*x*) were identified. The most common were pentaploids (53.6%) followed by hexaploids (22.7%) and tetraploids (21.6%). Higher cytotype diversity was found at lower latitudes than at higher latitudes (>52° N), where only tetraploids and pentaploids occurred. We detected 17.4% of mixed-ploidy populations, usually as a combination of two, rarely of three, cytotypes. The majority of mixed-ploidy populations were found in zones of sympatry of the participating cytotypes, suggesting they have arisen through migration (secondary contact zone). Using coarse-grained variables (climate, soil), we found evidence of both niche expansion and innovation in tetraploids related to triploids, whereas higher ploidy levels showed almost zero niche expansion, but a trend of increased niche unfilling of tetraploids. Niche unfilling in higher ploidy levels was caused by a contraction of niche envelopes toward lower continentality of the climate and resulted in a gradual decrease of niche breadth and a gradual shift in niche optima. Field-recorded data indicated wide habitat breadth of tetraploids and pentaploids, but also a pattern of increasing synanthropy in higher ploidy levels. Wide niche breadth of tetra- and pentaploids might be related to their multiple origins from different environmental conditions, higher “age”, and retained sexuality, which likely preserve their adaptive potential. In contrast, other cytotypes with narrower niches are mostly asexual, probably originating from a limited range of contrasting environments. Persistence of local ploidy mixtures could be enabled by the perenniality of *A. oleraceum* and its prevalence of vegetative reproduction, facilitating the establishment and decreasing exclusion of minority cytotype due to its reproductive costs. Vegetative reproduction might also significantly accelerate colonization of new areas, including recolonization of previously glaciated areas.

## Introduction

Polyploidy is a widespread phenomenon among flowering plants (e.g., Wendel, 2000; Van de Peer et al., 2017). Several lines of evidence suggest that all flowering plants have experienced several polyploid events at some points in their ancestry (Wood et al., 2009; Jiao et al., 2011), and polyploidization is also an active evolutionary process in many lineages (Soltis and Soltis, 2012; Soltis et al., 2016; Levin, 2020). Before becoming evolutionarily successful, newly formed polyploids often have to overcome numerical inferiority, mating incompatibility, and competition with parents (Levin, 1975). Polyploidization has profound consequences for the physiological and ecological behavior of plants (Levin, 2002; Soltis et al., 2004; Ramsey, 2011; Ramsey and Ramsey, 2014) as well as their genetic diversity (Soltis and Soltis, 2000). Interactions between nucleotypic effect (Bennett and Smith, 1972), increased genetic buffering, and changes in gene expression in polyploids (Adams and Wendel, 2005; Yoo et al., 2014; Gallagher et al., 2016) may drive phenotypic changes which not only immediately affect the ecology of polyploids (Ramsey, 2011), but also their potential for novel adaptive responses to selection (Bretagnole and Thompson, 1996; Otto and Whitton, 2000; Levin, 2002; Soltis et al., 2004; Balao et al., 2011; Visger et al., 2016). Niche separation is highly important in the establishment and further spread of neopolyploids (Fowler and Levin, 2016), decreasing intercytotype reproductive contacts and competition, thus increasing neopolyploid population growth (Ramsey and Schemske, 1998; Rieseberg and Willis, 2007). Where (two) divergent genomes merge, allopolyploids may exhibit even greater rates of population establishment, persistence, and exploitation of novel habitats than autopolyploids (Arrigo et al., 2016; Barker et al., 2016; Solhaug et al., 2016) due to exceeding parental niches caused by great flexibility in gene expression (Doyle et al., 2008; Leitch and Leitch, 2008; Yoo et al., 2014). Indeed, it has been evidenced repeatedly that polyploids (prevalently tetraploids) have a broader niche or may differ in their niche optima from their diploid progenitors (Soltis and Soltis, 1995, 2000; Levin, 2002; Weiss-Schneeweiss et al., 2013; Ramsey and Ramsey, 2014; Baniaga et al., 2020). They are reported to have a stronger colonization ability (Treier et al., 2009), including invasion potential (Pandit et al., 2006, 2011), and increased ability to cope with environmental extremes better, especially at higher latitudes and elevations, and in arid or artificially disturbed habitats (Grant, 1981; Stebbins, 1984; Brochmann et al., 2004; Wu et al., 2010; Ramsey, 2011; Manzaneda et al., 2012; te Beest et al., 2012; Muñoz-Pajares et al., 2018; Rice et al., 2019; Castro et al., 2020; Decanter et al., 2020).

The study of niche changes has recently made rapid progress incorporating readily available large-scale climatic data (Fick and Hijmans, 2017) and developed new statistical tools (Warren et al., 2008; Broennimann et al., 2012; Guisan et al., 2014). Several studies examining ecological (usually climatic) differentiation between polyploids and their progenitors within groups of closely related taxa do, however, not always support niche innovation in polyploids (e.g., Broennimann et al., 2007; Godsoe et al., 2013; Theodoridis et al., 2013; Glennon et al., 2014; Arrigo et al., 2016; Marchant et al., 2016). These results suggest substantial controversy in this area, which might be explainable partly by methodological incongruities between studies due to the use of a too coarse resolution of studied abiotic variables (Kirchheimer et al., 2016), ignorance of other axes of a taxon's niche (Guisan et al., 2014; Guignard et al., 2016; Segraves and Anneberg, 2016) or absence of assessments of niche differentiation of cytotypes across more spatial scales (Treier et al., 2009; Laport et al., 2013; Čertner et al., 2019). Apart from the methodological issues, niche evolution in polyploids may also be affected by an ancestral niche breadth of their progenitors and “age”, order or number of cytotype origins within polyploid series (Theodoridis et al., 2013; López-Jurado et al., 2019). Higher ploidy levels, in contrast to lower ones, may show a lower probability of niche expansion (Brittingham et al., 2018; López-Jurado et al., 2019) because they may attain their ecological limits during the filling of remaining available unoccupied niche space (Araújo et al., 2013) and their larger genome sizes might constrain their adaptive ability (Pandit et al., 2014). On the other hand, multiple origins of polyploids (Soltis and Soltis, 1999) might increase the genetic and physiological diversity of cytotypes which subsequently enhance their ecological tolerances (McIntyre, 2012; Karunarathne et al., 2018; López-Jurado et al., 2019).

Existence of diverse global and local distribution patterns of cytotypes within polyploid complexes, ranging from sympatry through more common parapatry with cytotype-mixed populations over contact zones to allopatry (Lewis, 1980; reviewed by Stebbins, 1985; Thompson and Lumaret, 1992; Petit et al., 1999; Levin, 2002; Martin and Husband, 2009; Husband et al., 2013; Kolář et al., 2017) suggest that cytotype distributions are the results of complex processes and that certain distribution patterns (e.g., local sympatry, large-scale allopatry) can be generated by processes other than niche evolution (Šingliarová et al., 2019; Wos et al., 2019) or that some of these processes (e.g., reproductive isolation) could reinforce niche differentiation between cytotypes (Rausch and Morgan, 2005; Rojas-Andrés et al., 2020). Modification of the reproductive system toward autogamy, apomixis, or vegetative propagation to secure reproduction in polyploids (Barringer, 2007; Paule et al., 2011; Herben et al., 2017) can be a key advantage in their local establishment irrespective of their initial minority status (Kao, 2007, 2008). Asexual reproduction drastically affects the dispersal abilities of polyploids, allowing their rapid colonization and establishment in new areas, which are particularly important when resource competition with an ancestor excludes them from local populations (Baker, 1967; Kearney, 2005; Kirchheimer et al., 2016). If residual sexuality is retained in the polyploid, it also preserves its adaptive potential (Cosendai et al., 2013). However, the same distributional patterns may also be explained as the results of stochastic processes, e.g., founder and drift effects (Lewis, 1967; Kliber and Eckert, 2005; Kolář et al., 2016). The present distribution of cytotypes may also mirror the position of past cytotype refuges in relation to sites available for colonization by single cytotypes, e.g., after ice retreat during the Quaternary (van Dijk et al., 1992; Mandáková and Münzbergová, 2006; Godsoe et al., 2013).

With the application of flow cytometry (FCM), which allows us to order more sample analyses, it has also become clear that mixed-ploidy populations are much more frequent than previously anticipated ones (e.g., Halverson et al., 2008; Cires et al., 2010; Marhold et al., 2010; Trávníček et al., 2010, 2011a,b; Čertner et al., 2017, 2019). In the past, cases of locally coexisting cytotypes were assumed to represent transient situations following the frequent generation or, in the case of secondary contacts, immigration of an alternative cytotype (Kao, 2007). However, commonly occurring cytotype-mixed populations intermixed with single-cytotype populations extending over large areas have been detected in several species (Husband et al., 2013; Kolář et al., 2017 and references therein). The knowledge of cytotype composition within mixed-cytotype populations and their spatial context with cytotype-uniform populations could serve as a basis for addressing questions of the frequency of polyploid formation, niche differentiation of cytotypes, and polyploid evolution (Husband et al., 2013). However, only little is known about many polyploid complexes because they have not been studied systematically throughout their entire distribution range (Afonso et al., 2020).

An example of a mixed-ploidy plant with a complex cytogeographic pattern at various spatial scales is *Allium oleraceum* L., consisting of tri-, tetra-, penta-, hexa-, hepta-, and octoploid cytotypes (2*n* = 24, 32, 40, 48, 56, 64) of presumably allopolyploid origin (Duchoslav et al., 2010, 2013). Detailed screening of its cytotype distribution in Central Europe has demonstrated complex spatial patterns, ranging from parapatry to sympatry at the landscape scale, with frequent occurrence of mixed-ploidy populations representing mostly secondary contacts between cytotypes (Duchoslav et al., 2010; Šafářová and Duchoslav, 2010; Šafářová et al., 2011), This complex cytogeographic pattern was explained by the interaction of several mechanisms, including (i) observational (Duchoslav et al., 2010; Šafářová et al., 2011) and experimental evidence (Ježilová et al., 2015; Duchoslav et al., 2017) for slight niche differentiation among cytotypes, (ii) the prevalence of asexual reproduction via aerial bulbils (Fialová and Duchoslav, 2014; Fialová et al., 2014), facilitating founder events and escape from minority cytotype exclusion effect (Levin, 1975) at mixed-cytotype sites, supported also by (iii) unstable environmental conditions at many sites caused by periodic disturbances and local patterns of migration in strongly human-influenced landscapes (Duchoslav et al., 2010). However, there are no robust data on cytotype diversity, distribution patterns and niche differentiation at a large spatial (continental) scale which could differ from those at a regional scale due to an interaction of large-scale (climate) and small-scale environmental factors (Treier et al., 2009; Laport et al., 2013; Čertner et al., 2019) and/or historical causes (te Beest et al., 2012). *Allium oleraceum* is distributed (Meusel et al., 1965) from the northern part of the Mediterranean basin, a refugium of thermophilic and mesic plant species during Glacial periods (Médail and Diadema, 2009), where it is currently in contact with its supposed diploid progenitors of the *Allium paniculatum* L. group (Pastor and Valdés, 1983; Brullo et al., 1996a; Salmeri et al., 2016), to Northern Europe, which was glaciated during the Last Glacial Maximum (LGM; Vandenberghe et al., 2014). Therefore, the current distributional pattern of cytotypes may also have been affected by the interaction between the effects of Pleistocene climatic oscillations, which had a profound effect on range expansions, retraction, and melting of differentiated lineages in species of the European flora (Hewitt, 1999; Stewart and Lister, 2001; Stewart et al., 2010), on the one hand and ecological attributes of cytotypes on the other. Knowledge of cytotype composition of populations and cytotype distribution, especially over contact zones between *A. oleraceum* and its presupposed progenitors, may also allow for inferences about the evolutionary history of polyploidy in *A. oleraceum*.

Specifically, we addressed the following questions: (1) What is the diversity of *A. oleraceum* cytotypes and the pattern of their geographic distribution over the species range? Is there a different cytotype composition in the contact zones with presumed progenitors in comparison to northern, previously glaciated regions without such contacts? (2) Is the current cytotype distribution a consequence of niche divergence? Is there a different pattern of niche shift with increasing ploidy level? (3) How frequent are mixed-ploidy populations, which cytotypes participate in their composition, and what processes stand behind their existence?

## Materials and Methods

### Studied Species

*Allium oleraceum* is a geophyte with prolific asexual propagation by aerial bulbils formed within an inflorescence and partly also by daughter bulbs produced belowground by the mother bulb, while sexual seeds are formed less frequently and usually in small numbers (Fialová and Duchoslav, 2014; Fialová et al., 2014). It belongs to *Allium* section *Codonoprasum* Reichenb., an evolutionarily young group consisting of a set of diploid and polyploid species (Friesen et al., 2006). This section is distributed in Northern Africa and Europe, extending to Iran and southwestern Siberia (Vvedenskii, 1935; Meusel et al., 1965; Stearn, 1980; Brullo et al., 1996a,b; Brullo et al., 2001). *Allium oleraceum* shows morphological similarity to species of the informal *A. paniculatum* complex, reaching its northern range limit in the southern parts of Europe (Stearn, 1980; Pastor and Valdés, 1983; Brullo et al., 1996a, 1997, 2003; Dobrotchaeva et al., 1999; Ciocârlan, 2000; Jauzein and Tison, 2001; Bogdanović et al., 2008; Aedo, 2013; Tison and de Foucault, 2014; Ghendov, 2015; Salmeri et al., 2016; Brullo and Guarino, 2017), and characterized by plants with ribbed and glabrous leaves with a semicylindrical to flat outline, spathe valves with a long appendage, and a campanulate perigon with stamens included or just slightly exerted (Brullo et al., 1996a,b, 2001, 2003, 2008; Salmeri et al., 2016). The origin of *A. oleraceum* is still puzzling; nevertheless, an alloploid origin is the most probable (Levan, 1937; Vosa, 1976; Duchoslav et al., 2010).

### Plant Material and Field Sampling

For the chromosome number survey, data on chromosome counts of *A. oleraceum* was extracted from 33 publications resulting in 399 localities (Supplementary Table 1, including complete reference list). The dataset was supplemented with unpublished chromosome data of 20 populations sampled by Fialová (1996) throughout the Czech Republic. Unfortunately, only limited information concerning the number of analyzed individuals and habitat conditions of sampled sites was available from most published papers. For this reason, one individual per ploidy was considered within a population.

Plant material was sampled from 2004 to 2019 throughout Europe covering most of the range of *A. oleraceum*. Additional samples of seeds or bulbils originating from 20 natural populations were obtained via “Index seminum” from foreign botanical gardens. In total, material from 446 populations of *A. oleraceum* was collected. Simultaneously, data from the detailed ploidy-level screening of 418 populations of the Czech Republic and Slovakia (Duchoslav et al., 2010; Šafářová and Duchoslav, 2010; Šafářová et al., 2011) was extracted and incorporated into subsequent analyses. Altogether, data on the cytotype composition of 1,283 populations was collected (Supplementary Table 1).

Field sampling followed our previous studies (Duchoslav et al., 2010; Šafářová and Duchoslav, 2010; Šafářová et al., 2011) allowing for joint analyses of all datasets. Specifically, sampling was usually carried out during the spring season (March to early June) when both non-flowering and flowering plants are present aboveground (Duchoslav, 2009), and plants were collected from a wide spectrum of habitats to attain a maximum representation of the species' niche. Each population sample consisted of mostly 3–30 plants depending on population size. An effort was made to avoid collection of individuals growing close together and to cover the entire population. During sampling, the sampling area expressed in square meters was estimated. Plants were cultivated in the experimental garden of Palacký University Olomouc, Czech Republic. Herbarium specimens are deposited in the Herbarium of Palacký University Olomouc (OL).

### Flow Cytometry and Chromosome Counts

DNA ploidy levels (Suda et al., 2006) were determined using FCM with *Triticum aestivum* cv. *Saxana* (2C DNA = 34.24 pg, Šafářová and Duchoslav, 2010) as an internal standard. Measurements were conducted on the following flow cytometers using two different fluorochromes: (i) BD Accuri C6 (BD Biosciences, San Jose, CA, USA)—propidium iodide (PI); (ii) Partec PAS (Partec GmbH, Münster, Germany)—PI and DAPI (4,6-diamidino-2-phenylindole); (iii) Partec Cy Flow ML (Partec GmbH)—PI and DAPI staining. Samples were prepared following the simplified protocol with LB01 isolation buffer and stained with either PI or DAPI (Doležel et al., 2007). Fresh leaves or meristematic buds of bulbil were used for FCM. For each run, the fluorescence intensity of at least 3,000 particles was recorded. Mostly, each individual was analyzed separately, but sometimes 2–3 individuals from the same population were analyzed together. Generally, histograms (using both PI and DAPI) with a coefficient of variation (CV) <5.0% were accepted. The ploidy level of the sample was determined by the position of its G_0_/G_1_ peak relative to the G_0_/G_1_ peak of an internal standard (i.e., relative fluorescence). Ploidy level was assessed based on calibration using plants for which chromosome numbers were previously (Duchoslav et al., 2010, 2013) as well as newly counted, covering the studied geographic range. Chromosome numbers were counted following the protocol by Duchoslav et al. (2010). Several individuals were analyzed with both PI and DAPI to calibrate the position of peaks if a different dye was used. Several samples with a DNA content on the margin of variation of the respective ploidy level were additionally subjected to chromosome counting to detect possible aneuploidy.

### Coarse-Grained Environmental Data Extracted From Climatic and Soil Layers

Environmental data related to different eco-physiological constraints of the studied species were selected and downloaded from various open-source databases. Since climate is often seen as the main factor driving species distributions at large scales (Guisan and Thuiller, 2005; Rice et al., 2019), annual trends and extreme limiting conditions related to precipitation and temperature (BIO 1-19 variables) and elevation were extracted from WorldClim 2.1 (Fick and Hijmans, 2017). Also mean annual solar radiation (kW.m^−2^) was downloaded from this database. Two variables related to evapotranspiration processes and rainfall deficit for potential vegetative growth (Global Aridity Index and Potential Evapotranspiration) were downloaded from Global Aridity Index and Potential Evapotranspiration Climate Database v2 (Trabucco and Zomer, 2019). Since edaphic conditions might play an important part in a polyploid's niche (Šmarda et al., 2013; Guignard et al., 2016), available quantitative physical and chemical soil variables were downloaded from the SoilGrid database (Hengl et al., 2014, 2017). All downloaded variables had a resolution of 30 arcseconds (~1 km).

Since collinearity is a common feature in any descriptive ecological data set and can be a problem for parameter estimation potentially leading to the wrong identification of relevant predictors in a statistical model (Dormann et al., 2013), all downloaded environmental variables were examined for pairwise correlations based on extracted values of environmental variables for all established locations of *A. oleraceum*. After evaluation, 13 not highly correlated variables (Pearson's correlations ≤ 0.70) were retained and used in further analyses (Supplementary Table 2).

### Field-Recorded, Local-Scale Environmental Data

At each sampled site the following set of local environmental variables was recorded, adopting and revising those used in the previous study by Duchoslav et al. (2010): (i) Habitat type was assessed in the field and classified using the recent vegetation classification of Europe consistent with the Braun-Blanquet approach (Mucina et al., 2016). Because of the large geographic range and consecutively great vegetation diversity of the sampled area, ecologically similar vegetation types were merged into ten aggregated habitat types (rocky outcrop, alpine grassland, dry grassland, mesic grassland, seminatural dry forest, seminatural mesic forest, seminatural alluvial forest, shrub, *Robinia pseudacacia* forest, arable land & field margin), which were used for subsequent analyses (“habitat type”). Correspondence between aggregated habitat types and observed vegetation types is explained in the Supplementary Table 3; (ii) Habitat naturalness, i.e., the vegetation at the site was classified according to decreasing degree of anthropic impact (synanthropy) into one of three levels: (1) “highly human-affected” (HHA; vegetation strongly influenced by man, typically with a relatively high representation or dominance of ruderal species, e.g., intensively managed agricultural habitats and their margins, road ditches, urbanized areas), (2) “extensively human-affected” (EHA; extensively cultivated landscapes, cultivated and plantation-like forests and shrubs, partly synanthropic, and extensively cultivated locations) and (3) “natural” (NAT; natural and seminatural vegetation without strong anthropic influence, e.g., semi-natural forests, semi-natural grasslands, relict sites). (iii) Habitat heterogeneity, i.e., the number of aggregated habitat types inhabited by the local *Allium* population. (iv) Presence of arable land: the effect of agricultural practices on the potential dispersion of *Allium* propagules into a site was approximated by the distance of the population to the nearest arable field and transformed into two categories (0 = >20 m; 1 = ≤20 m). (v) Rock proximity: populations were classified into two categories according to their proximity to rocky outcrops as follows: 0/1 = absence/presence of rocky outcrops within circle of 100 m radius around the population. (vi) Light conditions were assessed in the field according to the visually estimated proportion of full sunlight falling on the ground during late spring according to the following ordinal scale: 1 = strong shade, 2 = half-shade, 3 = light shade, 4 = full insolation. (vii) Heat load, i.e., a unitless index estimating the amount of heat absorbed by local site from solar radiation. The heat load was calculated from the slope, aspect and latitude of the site, using equation 2 in McCune and Keon (2002). The final values were converted to an arithmetic scale with the exp(x) function. (viii) Elevation, recorded *in situ* with a GPS instrument.

### Calculation of Niche Characteristics in Environmental Space (E-Space) and Local-Scale Environmental Differences Between Cytotypes

Niche characteristics of the cytotypes were estimated with two groups of variables: (i) coarse-grained environmental (climatic and soil) data downloaded from open-source databases and (ii) field-recorded, local-scale environmental data. As the first step, georeferenced location data were spatially stratified to avoid discrepancies caused by highly unequal sampling in different parts of the species range, using R package “spThin” (Aiello-Lammens et al., 2015). Since octoploids were only found at two sites, these were excluded from all ecological analyses. Considering the extremely dense sampling in Central Europe (Czech Republic, Slovakia; Duchoslav et al., 2010; Šafářová et al., 2011), Finland (Åström et al., 2015), and Latvia (Karpavičienė, 2012), a 40-km threshold distance was used for each cytotype found in those regions. In the remaining parts of Europe, a 15-km threshold distance was used for each cytotype. This resulted in 560 localities, which were used for subsequent ecological analyses (Supplementary Table 1).

Environmental niche space occupied by each cytotype and quantification of niche overlap, equivalence, and similarity between cytotypes were accessed with an ordination technique (PCA-env) which applies kernel smoothers to the cytotype presences in environmental space for the selection, combination, and weighting of environmental variables (Broennimann et al., 2012). As to environmental data, 13 environmental variables from climatic and soil open-source databases were used (Supplementary Table 2). We specified a division of the environmental space of PCA (the first two axes) into a grid of 200 × 200 cells, in which each cell corresponds to a unique vector of the available environmental conditions in the background area, i.e., available environmental conditions from which each cytotype is presumed to select its habitat. It is recommended that the background area is taken from buffer zones around known occurrences or from range maps (McCormack et al., 2010; Warren et al., 2010). Therefore, we used a 20-km buffer zone around the occurrence points of each cytotype for the definition of a background area. The number of background (random) points per cytotype equalled a hundred-fold of the number of the respective occurrence points.

The niche overlap between each pair of cytotypes was computed employing the Schoener's D statistic (D) directly from environmental niche space (Schoener, 1968; Warren et al., 2008). The value of D ranges from 0, when two cytotypes have no overlap in the environmental space, to 1, when two cytotypes share the same environmental space. For testing niche conservatism vs. evolution, the niche equivalency (identity) test and the niche similarity test (Warren et al., 2008) were computed for each pair of cytotypes. The niche equivalency test determines whether niches of two cytotypes are equivalent, i.e., whether the niche overlap is constant when randomly reallocating cytotype identities over compared cytotype ranges (Broennimann et al., 2012). Specifically, occurrences of compared cytotypes were pooled and randomly split into two datasets, with the same sizes as the two original datasets, after which D was calculated. This process was repeated 1,000 times and generated a null distribution of D (Warren et al., 2008). We determined the non-equivalence or conservatism of environmental niches if the observed D of the cytotypes being compared were within the lower or upper 2.5% quantile, respectively, of a null distribution of simulated values of D.

The niche similarity test establishes whether the observed niche overlap between two cytotypes is different from the overlap between the observed niche of one cytotype and niches randomly selected from the available environmental background of the other cytotype within the environmental space defined by PCA axes (Broennimann et al., 2012). Specifically, points were randomly selected from the background area of one cytotype and the niche of this random sample was then compared to the observed niche of another cytotype using D. This process was repeated 1,000 times and generated a null distribution of D (Warren et al., 2008). If the observed D was within the upper or lower 2.5% quantile of a null distribution of simulated values of D, the cytotypes were considered to be more similar or more dissimilar, respectively, than expected by chance. This test was applied in both directions, i.e., by resampling the occurrences of the first cytotype and then those of the other cytotype.

Schoener's D statistic does not contain information on optima and breadths of niches (Glennon et al., 2014). Therefore, we used the procedure described in Theodoridis et al. (2013) and Kirchheimer et al. (2016) for comparing niches in terms of optima and breadths. Specifically, 100 cells were randomly resampled in the niche of each cytotype and their scores were extracted along the first two PCA axes. The niche optimum and the niche breadth were calculated as the mean and the variance of the sampled scores along the first and the second PCA axes. This procedure was repeated 1,000 times. Distributions of values of niche optimum and breadth for each PCA axis were compared between cytotypes.

Additionally, the following indices of niche change were computed relative to the niche of lower ploidy level only (Petitpierre et al., 2012; Guisan et al., 2014): niche expansion (E), i.e., proportion of the niche space of the higher ploidy level non-overlapping the niche of the lower ploidy level; niche unfilling (U), i.e., proportion of the niche space of the lower ploidy level non-overlapping the niche of the higher ploidy level; and niche stability (S_n_, S_e_), i.e., proportion of the niche of either lower (S_n_) or higher ploidy level (S_e_), shared with the other ploidy level. To decrease the effect of rare (marginal, extreme) environments on the estimation of the indices of niche change, analyses were performed in two settings, i.e., at the intersection of the 75th quantile and 95th quantile, respectively, of both compared environmental densities. All analyses were performed in the R platform (R Development Core Team, 2014), using the packages ecospat (Di Cola et al., 2017), raster (Hijmans et al., 2017), and ENMTools (Warren et al., 2010).

Ecological differentiation among cytotypes was also tested using eight field-recorded, local-scale environmental variables. Firstly, each environmental variable was used as the dependent variable and the ploidy level (with five cytotypes, 3*x*−7*x*) was used as the independent variable in separate univariate analyses. Mixed populations were duplicated according to the cytotype composition and duplicates were assigned in each group of participating cytotypes. Log-linear models were used for the analyses of categorical environmental variables, whereas the non-parametric Kruskal-Wallis test followed by multiple comparisons Dunn's test was used for the analyses of quantitative and ordinal variables (Zar, 1996). Subsequently, eight field-recorded environmental variables were subjected to constrained principal coordinate analysis (db-RDA; Legendre and Anderson, 1999) using the Gower coefficient of dissimilarity for mixed numeric/ordinal/categorical data (Legendre and Legendre, 1998). Due to the poor habitat description of 72 sites analyzed for niche characteristics in E-space, these sites were excluded from db-RDA, resulting in 488 analyzed populations (Supplementary Table 1). Cytotype composition of populations (**Table 2**) was used as the explanatory variable and visualized in an ordination diagram. Additionally, pairwise tests between cytotypes for environmental differences were performed using a reduced set of data matrices only consisting of populations of the respective pairs of cytotypes. Mixed populations were duplicated according to cytotype composition and duplicates were assigned to each group of participating cytotypes. Environmental differences between groups with different cytotype compositions were tested with a Monte Carlo permutation test using 999 permutations. The Bonferroni correction of α (at α = 0.05) for multiple tests was applied. Univariate analyses were performed using NCSS 9 (Hintze, 2013), whereas multivariate analyses were performed using CANOCO 5 (ter Braak and Šmilauer, 2012).

## Results

### Cytotype Diversity and Population Composition

Six ploidy levels ranging from tri- to octoploid (2*n* = 24, 32, 40, 48, 56, 64) were identified in our dataset using chromosome counting (Supplementary Figure 1). This was fairly consistent with the FCM analysis of 3,931 individuals from 446 European populations, newly analyzed in this study. Relative fluorescence of different cytotypes differed [Linear Models, PI dye: *F*_(5, 6.9)_ = 1630.5, *P* < 0.001; DAPI dye: *F*_(2, 4.7)_ = 97.2, *P* < 0.001; Table 1]. Large intracytotypic variation in relative fluorescence was observed in tetra- and pentaploids. This resulted in an almost continuous distribution of relative fluorescence values between tetra- and pentaploids (Supplementary Figure 2). However, both chromosome counts of several plants with marginal values of relative fluorescence (Supplementary Table 1) and spatial correlation of relative fluorescence values in tetra- and pentaploids (Supplementary Figure 2; see Duchoslav et al., 2013 for the identical pattern found for absolute DNA content) allowed us to infer the ploidy level of such plants with certainty. Moreover, no aneuploid counts were identified, not even in plants with marginal relative fluorescence values within particular cytotypes. This is in line with published data on karyologically examined plants from 399 localities (Supplementary Table 1) where only euploid chromosome counts (2*n* = 3*x*, 4*x*, 5*x*, 6*x*) were reported. The ploidy evaluation of a total of 11,163 individuals from 1,283 populations (incl. all previously published data; Supplementary Table 1) revealed that the most common cytotype was pentaploid (53.6%) followed by hexaploid (22.7%) and tetraploid (21.6%). Other ploidy levels, i.e., triploids (1.2%), heptaploids (0.8%), and octoploids (0.2%), were extremely rare.

**Table 1:** Relative fluorescence (RF) of the *A. oleraceum* cytotypes assessed using flow cytometry with the stain propidium iodide (PI) or 4,6-diamidino-2-phenylindole (DAPI).

**Ploidy level**	**PI**							**DAPI**						
	**Npop**	**Nind**	**Mean RF**	**SD**	**Min**	**Max**	**Variation (%)**	**Npop**	**Nind**	**Mean RF**	**SD**	**Min**	**Max**	**Variation (%)**
3*x*	16	132	1.25^a^	0.03	1.19	1.29	8.4	–	–	–	–	–	–	–
4*x*	134	2,087	1.56^b^	0.08	1.41	1.71	21.3	6	29	2.45^a^	0.09	2.36	2.59	9.7
5*x*	345	4,249	1.84^c^	0.05	1.66	1.98	19.3	36	286	2.84^b^	0.10	2.51	3.04	21.1
6*x*	89	1,858	2.10^d^	0.05	2.01	2.24	11.4	3	15	3.20^c^	0.06	3.16	3.27	3.4
7*x*	10	87	2.46^e^	0.06	2.36	2.52	6.8	–	–	–	–	–	–	–
8*x*	2	23	2.65^f^	0.05	2.62	2.69	2.7	–	–	–	–	–	–	–

*All values of relative fluorescence are calculated relative to the internal standard (Triticum aestivum cv. Saxana). Mean RF values within a column with different letters are significantly different at P ≤ 0.05 (Bonferroni multiple comparison test). All available data of RF measured by us (Supplementary Table 1) were used for calculations. Npop, number of populations analyzed; Nind, number of individuals analyzed; SD, standard deviation; Variation (%) = (Max-Min) × 100/Min*.

Considering populations with at least two individuals analyzed (858 populations, 97.1% of all populations sampled by us; the number of analyzed plants per population: mean ± SD, 13 ± 14, min. = 2, max. = 213), a high diversity in cytotype composition was found within populations (Table 2). The majority of populations comprised a single cytotype (83.6%), much fewer populations contained two (16.5%), and just eight populations (0.9%) contained three cytotypes. Among populations consisting of a single ploidy level, pentaploids were found to be the most common while octoploids were the rarest (Table 2).

**Table 2:** Cytotype composition of 858 populations of *Allium oleraceum* with at least two sampled individuals, and odds of uniform-ploidy populations (Odds), expressed as the ratio of the number of uniform-ploidy populations to the number of mixed-ploidy populations containing a particular cytotype.

**Population cytotype**	**Number of**	**Percent of**
**composition (Odds)**	**populations**	**total**
3*x* (4.0)	12	1.40%
4*x* (1.6)	114	13.28%
5*x* (3.3)	450	52.45%
6*x* (1.3)	123	14.33%
7*x* (4.5)	9	1.05%
8*x* (1.0)	1	0.12%
3*x* + 4x	1	0.12%
3*x* + 5*x*	2	0.23%
4*x* + 5*x*	51	5.94%
4*x* + 6*x*	10	1.17%
4*x* + 7*x*	1	0.12%
4*x* + 8*x*	1	0.12%
5*x* + 6*x*	74	8.62%
5*x* + 7*x*	1	0.12%
4*x* + 5*x* + 6*x*	8	0.93%

*Populations are grouped according to the number of cytotypes per population (one, two, or three). Frequencies are shown both in absolute numbers and in percentages of the total number of populations*.

More individuals were analyzed from populations revealed as mixed-ploidy (median = 15) than uniform-ploidy (median = 8) by FCM (Mann-Whitney one-sided test, z = 8.9, P < 0.001). Also a sampling area of mixed-ploidy populations (median = 300 m^2^) was larger than that of uniform-ploidy populations (median = 80 m^2^; z = 7.5, P < 0.001). However, habitat heterogeneity was similar between uniform and mixed-ploidy populations (z = 1.1, P = 0.286) and most populations inhabited a single habitat.

The proportion of mixed-ploidy populations was cytotype-dependent. Populations containing odd ploidy level(s) were uniform two to three times more likely than those containing even ploidy level(s) (Table 2). Among populations consisting of two cytotypes, 4*x* + 5*x* and 5*x* + 6*x* combinations were most common. On the other hand, only three mixed-ploidy populations consisting of triploids were found, once with tetraploids and twice with pentaploids. Heptaploids also rarely formed mixed-ploidy populations, once with tetraploids and once with pentaploids. Only one mixed-ploidy population consisting of tetraploids and octoploids was found. Mixed-ploidy populations comprising three cytotypes withal had the combination of 4*x* + 5*x* + 6*x*. Populations containing four or more cytotypes were not found (Table 2).

### Geographic Distribution of Cytotypes

**Figure 1:**
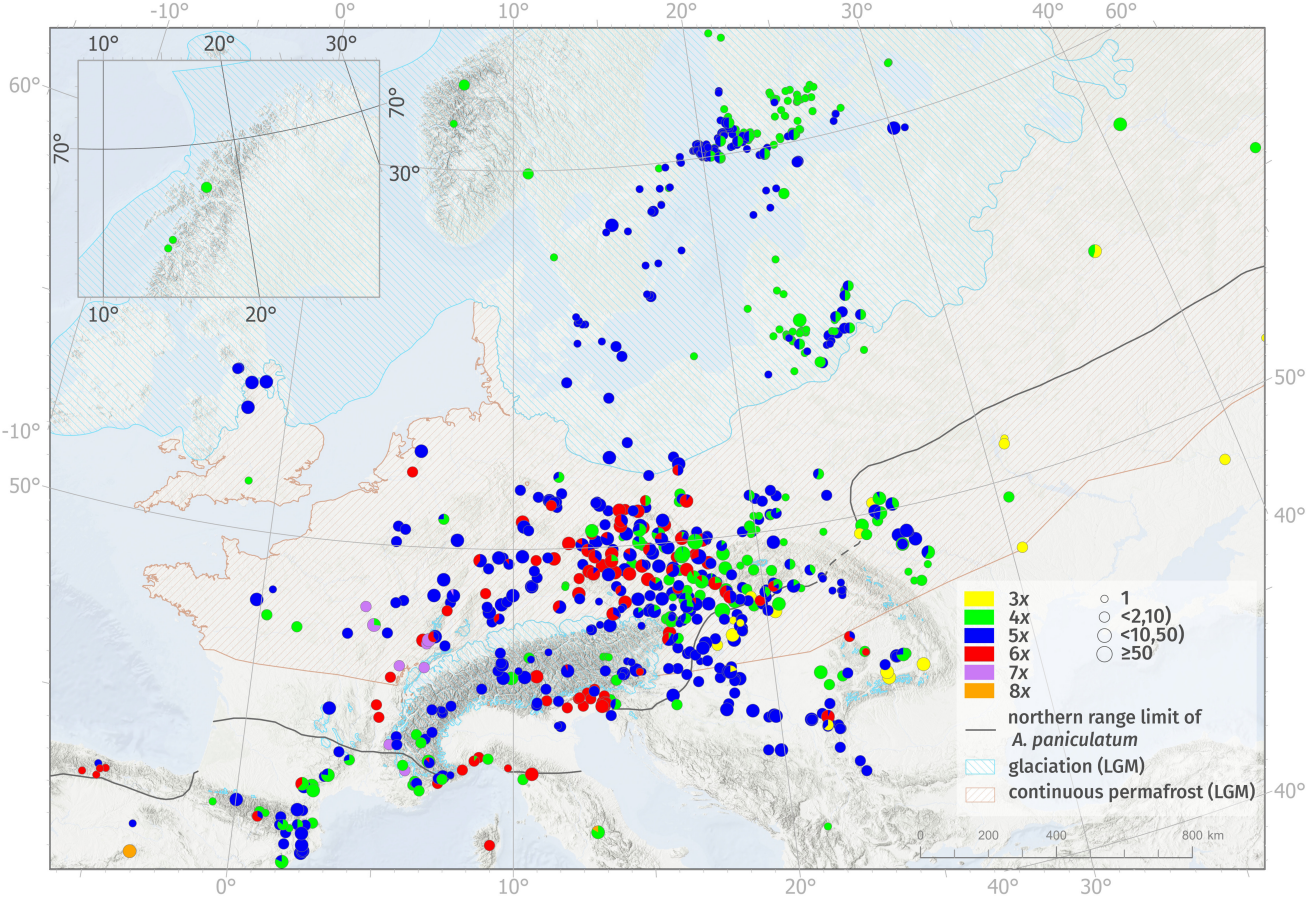
Geographic distribution of ploidy levels of Allium oleraceum, based on all available data (Supplementary Table 1). Different ploidy levels are distinguished by different colors: 3x—yellow, 4x—green, 5x—blue, 6x—red, 7x—purple, 8x—orange. Ploidy-mixed populations are depicted as a pie chart showing the local frequency of cytotypes. The size of the circle represents the number of analyzed individuals per population. For the sake of clarity, populations from the Czech Republic and Slovakia were thinned. Lines with hatching indicate the limits of the continuous permafrost (light brown line) and glaciation (light blue line or polygons) during the last permafrost maximum according to Lindgren et al. (2016). The inset map shows the distribution of cytotypes in northern Norway. The black line shows the northern range limit of diploids of the Allium paniculatum complex, compiled from various sources (see Materials and Methods).

Cytotypes differed considerably in their distribution patterns at both large and small spatial scales and also in intensity of spatial intermingling (Figure 1, Table 2, Supplementary Figures 3, 4). Cytotype diversity over the studied area was found to be higher at lower latitudes than at higher latitudes (>52°), where only tetra- and pentaploids occurred. This pattern roughly traces the zones without and with continuous glaciation during the LGM along with areas with and without the current occurrence of diploid taxa of the *A. paniculatum* complex (Figure 1, Supplementary Figures 3, 4).

Triploids were found to be restricted to the south-eastern part of Central Europe (Pannonian Basin and Transylvanian Basin) and Eastern Europe (Ukraine, southern part of European Russia) where they co-occurred in a mosaic-like pattern with tetra- and pentaploids. Specifically, two contact zones consist of (i) uniform tri- and pentaploid populations and mixed 3*x* + 5*x* populations in Hungary, and (ii) uniform tri- and tetraploid populations and one mixed 3*x* + 4*x* population in Russia, respectively. Moreover, two triploid populations were found in the contact zone with tetra-, penta- and hexaploids, one in southern Slovakia (for details see Šafářová et al., 2011), and another in southwestern Romania (Figure 1, Supplementary Figure 4).

Both tetra- and pentaploids were widespread and their distributions largely overlapped. Tetra- and pentaploid cytotypes formed a diffuse mosaic-like contact zone with both single-cytotype and mixed-ploidy populations over most of their ranges. Tetraploids had the largest geographic range of all cytotypes, from northern Spain to the coastal areas of northern Norway and Iceland (here as a non-native plant; Åström et al., 2015) and the European part of Russia. Pentaploids occurred more frequently in the western and central parts of Europe, reaching their eastern distribution limit along a line from western Ukraine to the eastern coastal areas of the Baltic Sea. At the landscape scale, however, we found several regions where tetra- and pentaploids were spatially segregated and formed ploidy-uniform areas (i.e., large-scale parapatry) with more or less pronounced contact zones with ploidy mixtures (e.g., the western part of Hungary, continuing southwards to Croatia and Serbia vs. Slovakia; Germany; Sweden vs. Finland; Figure 1).

Hexaploids were geographically restricted to Central Europe and northern parts of the west-Mediterranean area. A striking boundary of hexaploid distribution runs alongside the outer/inner ranges of the Western Carpathians and continues along the outer part of the Eastern Alps to southern Austria and northeastern Italy. Three isolated hexaploid populations (one single-cytotype and two mixed-ploidy populations) were found in Romania. Considering mixed populations consisting of hexaploids, only 5*x* + 6*x* mixed populations were found frequently and occurred in the contact zone of penta- and hexaploids, while 4*x* + 6*x* populations were rare, and with two exceptions, occurred only in Central Europe. Mixed 4*x* + 5*x* + 6*x* populations were found only in Central Europe, where tetra-, penta-, and hexaploid cytotypes co-occur. At the landscape scale, however, we detected several regions where hexaploids occupied ploidy-uniform areas (e.g., part of southern Bohemia in the Czech Republic, lowlands of the Friuli-Venezia Giulia and Veneto regions in northern Italy; Figure 1).

Heptaploids (incl. uniform and mixed populations 4*x* + 7*x* and 5*x* + 7*x*) were found only in the western foothills of the Western Alps and Jura in eastern and southeastern France and western Switzerland. Only two octoploid populations were discovered: one, ploidy-uniform, in central Spain, and the other, mixed with tetraploids, in central Italy (Figure 1).

### Distribution of Cytotypes Along Coarse-Grained Ecological Gradients

**Figure 2:**
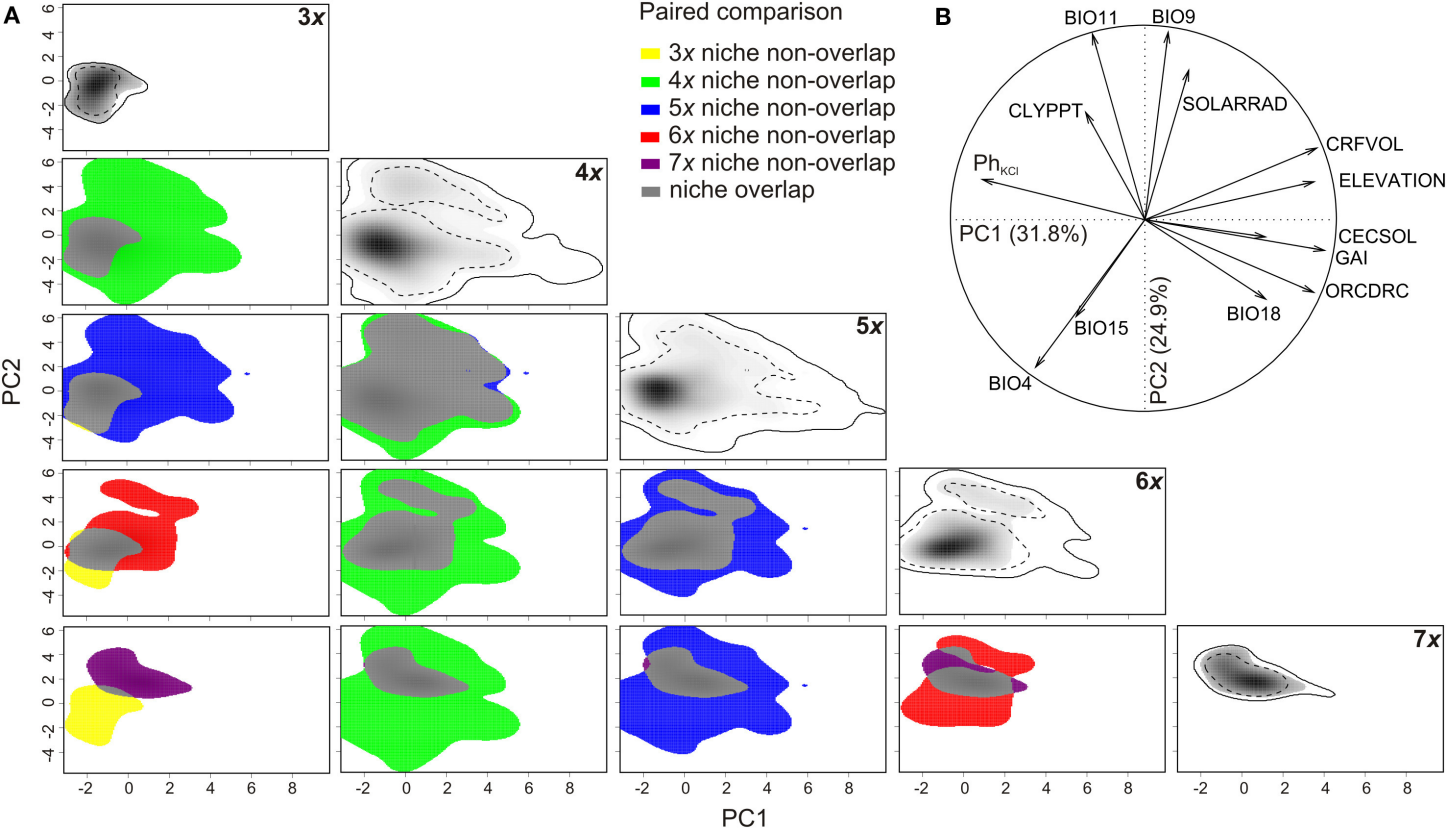
Niches of five cytotypes of Allium oleraceum in the environmental space of coarse-grained variables (climate, soil) along the first two axes of PCA (PCAenv). **(A)** The panels along the diagonal represent the niches of the respective cytotypes (3x−7x). The panels below the diagonal compare niches of all pairs of cytotypes. Niches of tri-, tetra-, penta-, hexa-, and heptaploids not overlapping the niche of the other cytotypes in pairwise comparisons are shown in yellow, green, blue, red, and purple, respectively. Niche overlap is shown in gray. Color shading shows the density of the occurrences of the cytotype. Full and dashed contour lines illustrate 100 and 50%, respectively, of available (background) environments delimited by a 20-km buffer zone around the occurrence points of each cytotype. **(B)** The correlation circle shows the loadings of the individual environmental variables to the first two PCA axes. BIO4, temperature seasonality; BIO9, mean temperature of driest quarter; BIO11, mean temperature of coldest quarter; BIO15, precipitation seasonality; BIO18, precipitation of warmest quarter; CECSOL, cation exchange capacity of soil [mmol(c)/kg]; CLYPPT, weight percentage of clay particles (<0.0002 mm); CRFVOL, volumetric percentage of coarse fragments (>2 mm); GAI, global aridity index; ORCDRC, soil organic carbon content; Ph_KCl_, soil acidity measured in KCl solution; SOLARRAD, mean annual solar radiation (kW.m^−2^).

The variation in available environmental conditions in the species range was summarized by two PCA axes, explaining 31.7% and 24.9%, respectively, of the total variation in the environmental space (Figure 2). The PC1 axis mirrored a general humidity gradient (in terms of increasing humidity expressed by the global aridity index and increasing precipitation in the warmest period of the year), associated with more acid, coarse-grained soils containing more soil organic carbon and a higher cation exchange capacity at higher elevations. The axis constrained the distribution of all cytotypes in the most humid climates with acid and coarse-grain soils, usually at higher elevations (Figure 2B). The triploids occupied an extreme position on the PC1 axis, corresponding to habitats with alkaline, fine-grained soils in more arid conditions, while other cytotypes were slightly shifted into more humid conditions, with heptaploids being found in the relatively most humid conditions. Both tetra- and pentaploids occupied a wide range of environmental conditions along PC1. The PC2 axis mirrored a general seasonality (continentality) gradient, in terms of increasing mean temperatures of both the coldest and the driest periods of the year, and decreasing temperature and precipitation seasonalities along PC2. Considering all cytotypes together, almost all available environmental conditions along PC2 were covered. However, the axis mainly constrained the distribution of the tri- and heptaploid cytotypes, for which it showed the highest differences in temperature and precipitation seasonalities. On the other hand, tetraploids, and to a lesser extent also penta- and hexaploids, covered a wide range of environmental conditions along PC2, with a tendency for two optima (dominant higher and less apparent lower climate seasonality) (Figure 2A).

Values of niche overlap (Schoener's D) between cytotypes ranged from 0.056 for tri- and heptaploids to 0.806 for tetra- and pentaploids, and usually exceeded 0.250, suggesting a moderate to high niche overlap. Results of niche equivalency tests were inconclusive in most cases but suggested that most of the niches are equivalent (Table 3; Figure 2A). For some pairs of cytotypes, the niches were significantly less (3*x*−7*x*) or more equivalent (4*x*−5*x*) than expected by chance. In the analysis of niche similarity, the majority of tests indicated insufficient power to make inferences regarding niche differentiation. For some pairs of cytotypes (3*x*−4*x*, 4*x*−5*x*, 5*x*−6*x*), the niche similarities were significantly higher than expected by chance, irrespective of the direction of the test.

**Table 3:** Niche overlap metric (Schoener's D) and results of niche equivalency tests, niche similarity tests, and indices of niche change in E-space (PCAenv) for five cytotypes of A. oleraceum.

**Allium** **cytotypes**		**Niche overlap (D)**	**Niche equivalency**	**Niche similarity**		**Indices of niche change (95th percentile)**			
**1**	**2**			**1 → 2**	**2 → 1**	**Expansion (E)**	**Stability (S_**e**_)**	**Unfilling (U)**	**Stability (S_**n**_)**
3x	4x	0.161	ns	More similar	More similar	0.101	0.899	0.000	1.000
	5x	0.227	ns	ns	ns	0.068	0.932	0.005	0.995
	6x	0.264	ns	ns	ns	0.077	0.923	0.107	0.893
	7x	0.056	Less equivalent	ns	ns	0.000	1.000	0.509	0.491
4x	5x	0.806	More equivalent	More similar	More similar	0.001	0.999	0.039	0.961
	6x	0.439	ns	ns	ns	0.000	1.000	0.232	0.768
	7x	0.224	ns	ns	ns	0.001	0.999	0.254	0.746
5x	6x	0.540	ns	More similar	More similar	0.000	1.000	0.145	0.855
	7x	0.251	ns	ns	ns	0.012	0.988	0.258	0.742
6x	7x	0.371	ns	ns	ns	0.234	0.766	0.243	0.757

*The niche similarity test was repeated in both directions, i.e., by resampling first the occurrences of higher ploidy level and then those of lower ploidy level. Expansion (E) and unfilling (U) indices represent proportions of the niche of higher ploidy level which is not occupied by the lower ploidy level and vice versa. Niche stability (S_n_, S_e_) represents the proportion of the niche of either lower (S_n_) or higher ploidy level (S_e_) shared with the other cytotype. Indices of niche change were calculated at the intersection of the 95th percentile of both compared environmental densities. We determined significance of niche equivalency and niche similarity tests if the observed D of the cytotypes being compared was within the lower (“less equivalent/similar”) or upper 2.5% (“more equivalent/similar”) quantile of a null distribution of simulated values of D, respectively (ns, non-significant)*.

Indices of niche change comparing higher vs. lower ploidy levels showed complex patterns (Table 3, Figure 2A). Particularly tetraploids showed the highest niche expansion and complete filling of the niche of triploids of all cytotypes. Higher ploidy levels showed decreasing niche expansion but slightly increasing niche unfilling of the triploid niche when compared with the pattern observed in the tetraploids. When comparing with the niches of tetraploids or pentaploids, niches of higher ploidy levels showed almost zero expansion but an increase in niche unfilling. Only heptaploids showed both higher niche expansion and higher niche unfilling when compared with the niche of hexaploids. Using a more stringent setting (i.e., removing more marginal environments; 75th percentile) resulted in qualitatively the same conclusions as in the case of a less stringent setting (95th percentile) (data not shown).

**Figure 3:**
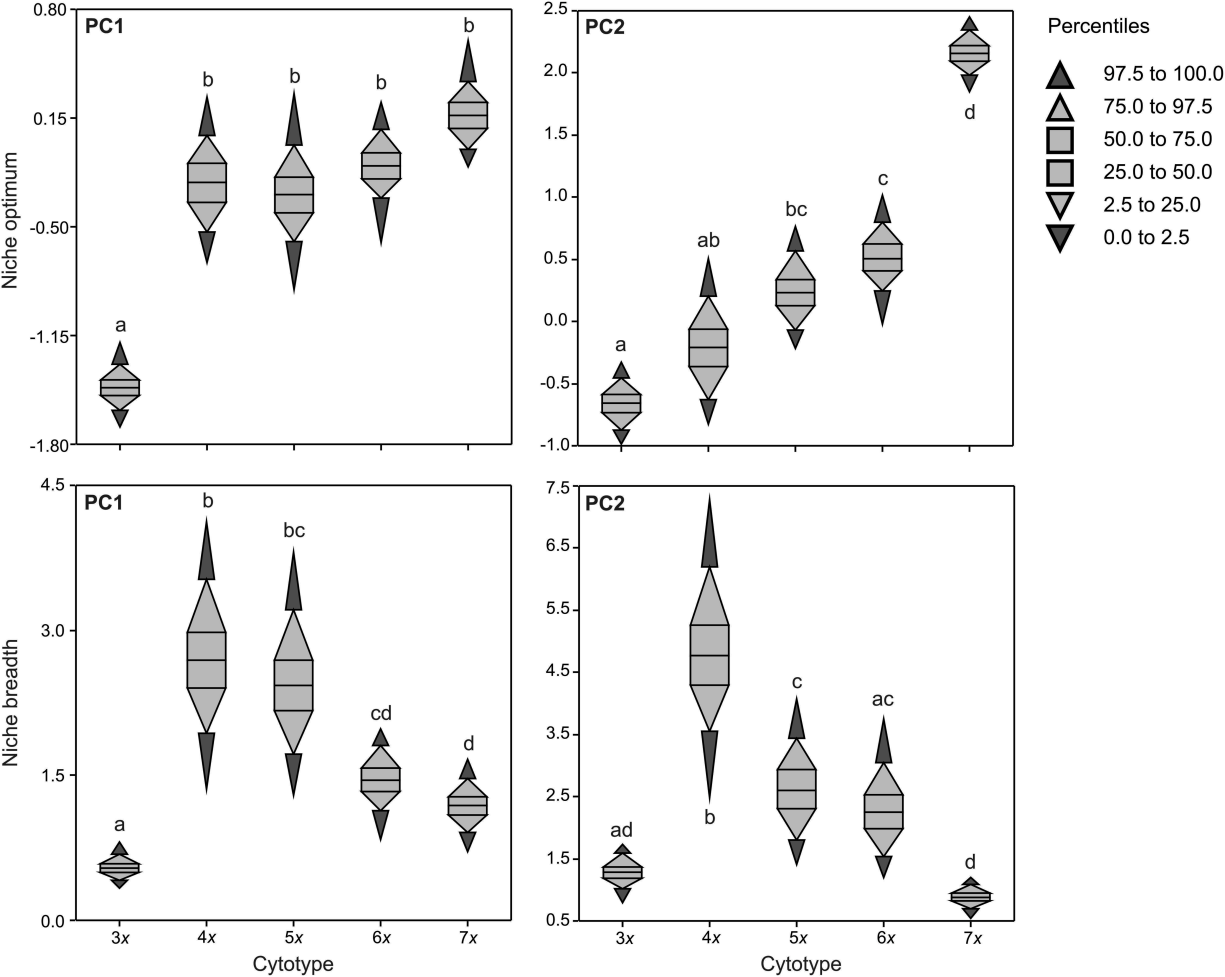
Niche optima and niche breadths of five cytotypes of *Allium oleraceum* along the first two PCA axes (PCAenv). The same letters in each panel indicate non-significant differences in niche traits between respective cytotypes (i.e. overlaps of 95% central ranges of cytotypes).

The niche optimum of triploids along PC1 was significantly different from that of other cytotypes, being shifted to the left. The niche optimum of cytotypes along PC2 significantly increased with increasing ploidy level, but there were also some overlaps in niche optima between nearby cytotypes (e.g., 3*x*−4*x*, 4*x*−5*x*, 5*x*−6*x*; Figure 3). Cytotypes differed in niche breadth along both PCA axes in a complex manner. Tetra- and pentaploids, and tetraploids showed the largest niche breadth along PC1 and PC2, respectively. Both lower (3*x*) and higher (5*x* in the case of PC2, 6*x*, 7*x*) ploidy levels had lower niche breadths than tetraploids, with triploids and tri- and heptaploids having the narrowest niches along PC1 and PC2, respectively (Figure 3).

### Local-Scale Environmental Differences Between Cytotypes

**Figure 4:**
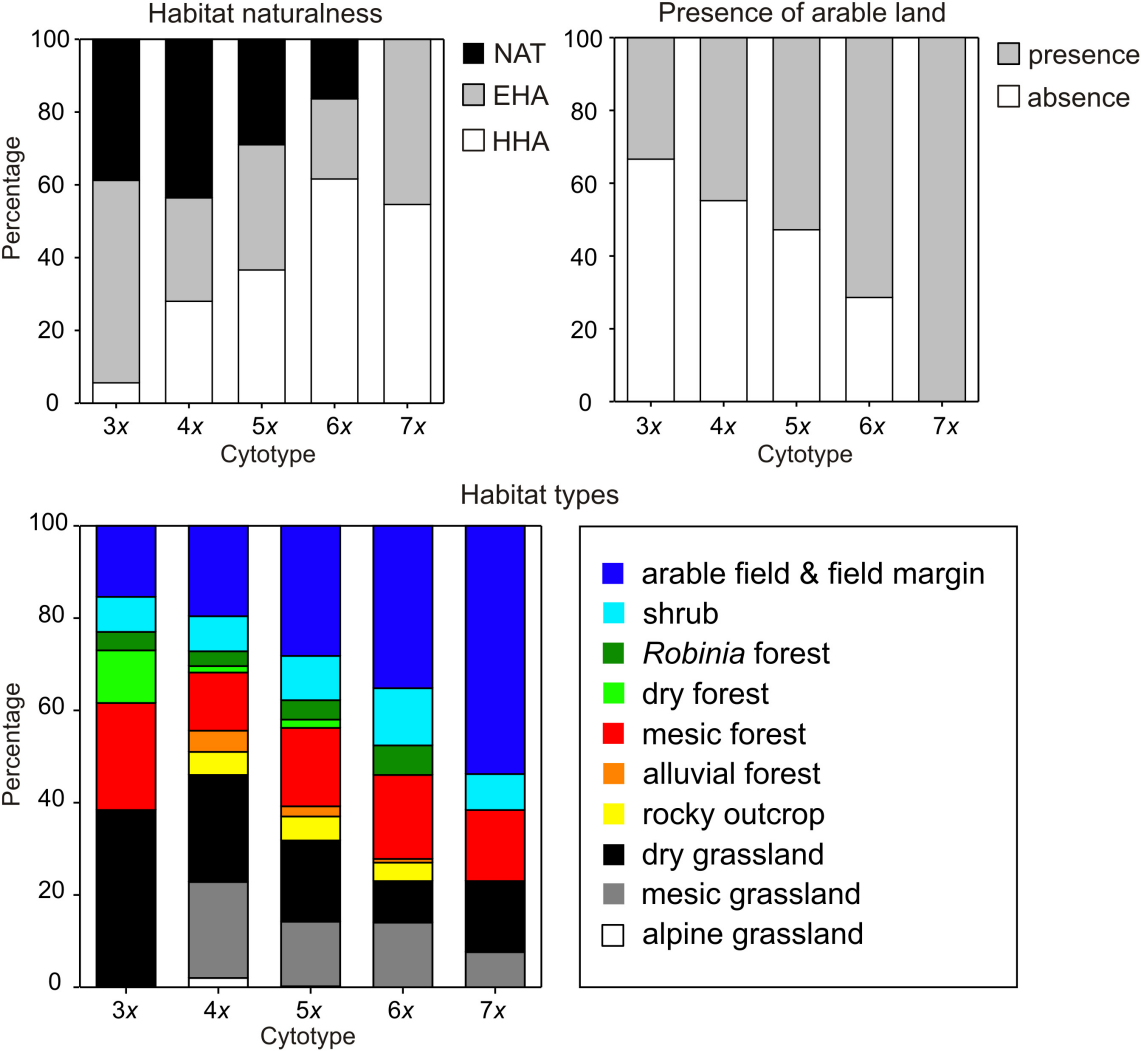
Frequency distribution (%) of categories of field-recorded environmental variables at sites of different cytotypes of *Allium oleraceum*: habitat naturalness (HHA, highly human-affected; EHA, extensively human-affected; NAT, natural habitats), presence of arable land (presence/absence: distance to the nearest arable field ≤/>20 m), and habitat types.

Cytotypes showed significantly different associations with particular habitat types although their habitat requirements overlapped (Table 4, Figure 4). Both tetra- and pentaploids were found in a wide spectrum of habitats, whereas tri- and heptaploids occupied a narrower spectrum of habitat types. Lower ploidy levels (3*x*−5*x*) were frequently found in open semi-natural habitats such as grassland and rocky outcrops, whereas higher ploidy levels (6*x*, 7*x*) were frequently observed in human-affected habitats such as arable land and field margins as well as *Robinia pseudoacacia* forests. Populations of higher ploidy levels occupied preferentially extensively to highly human-affected sites in close contact with arable land (Armitage test for trend in proportions, *z* = −4.81, *P* < 0.001; Figure 4). Populations of different cytotypes did not differ in habitat heterogeneity, light conditions of occupied habitats, and proximity of rocky habitats. Tetra- and pentaploids showed the widest elevation range from lowlands (0 m) to the alpine belt (c. 2,100–2,300 m), while tri- and heptaploids were found only at elevations below ca 600 m a s l. Hexaploids occupied slightly higher elevations than tetra- and pentaploids. Sites with the occurrence of tetraploids had a significantly lower heat load than sites of other cytotypes (Table 4, Supplementary Figure 5).

**Table 4:** Summary of the associations between cytotypes and field-recorded local-scale environmental variables in populations of *Allium oleraceum*.

**Variable**	**Test**	**DF**	**Test**	***P***
			**statistics**	
Habitat type	LLM	36	65.34	**0.002**
Presence of arable land	LLM	4	32.08	** <0.001**
Habitat naturalness	LLM	4	51.73	** <0.001**
Habitat heterogeneity	LLM	8	4.49	0.810
Rock proximity	LLM	4	13.04	0.011
Light conditions	K-W	4	5.66	0.226
Heat load	K-W	4	22.36	** <0.001**
Elevation	K-W	4	19.70	** <0.001**

*Differences were tested either by log-linear models (LLM) or Kruskal–Wallis test (K-W). P-values in bold are significant after Bonferroni correction (P <0.006)*.

**Figure 5:**
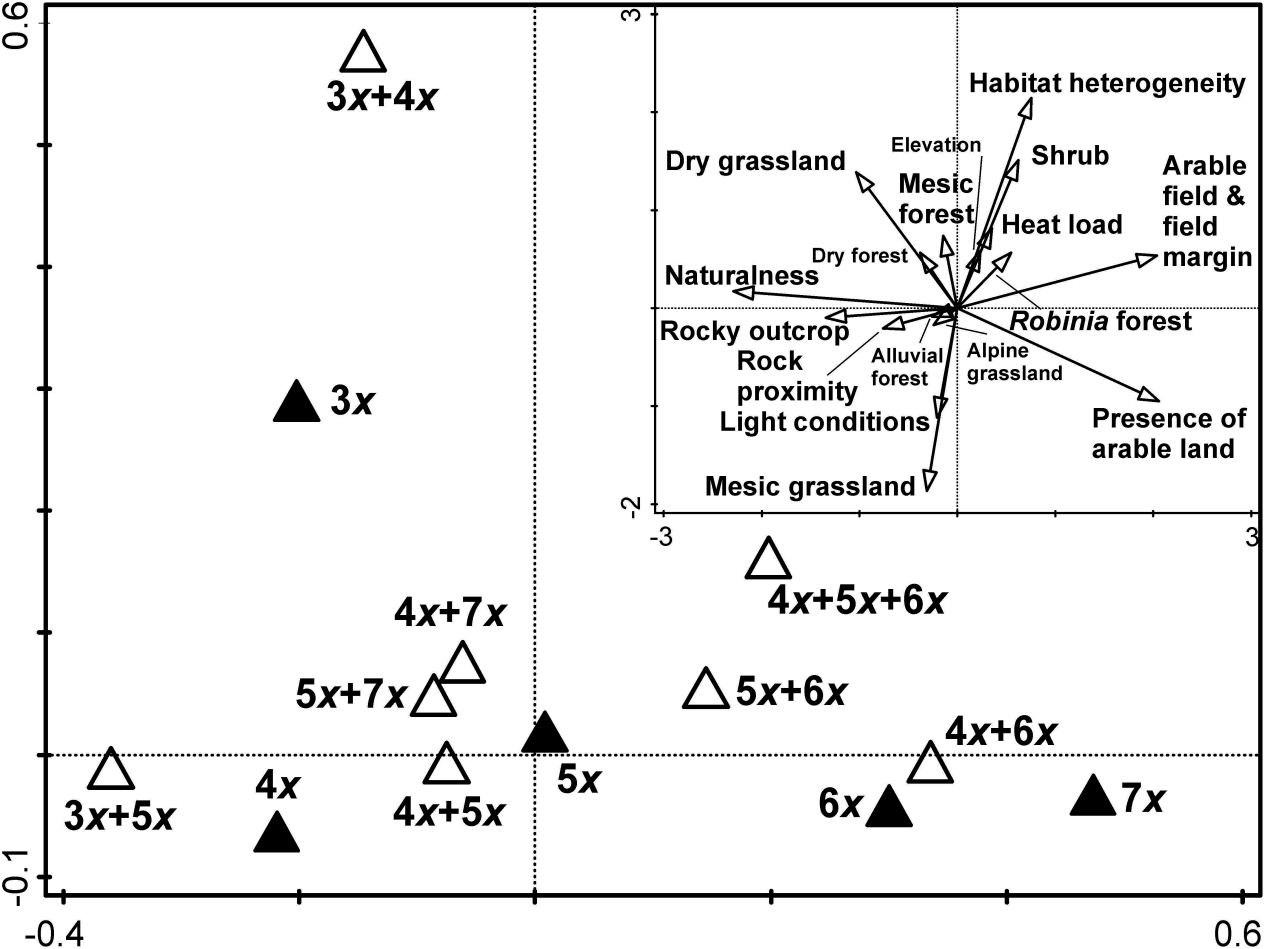
The first two canonical axes of constrained principal coordinate analysis (db-RDA). Cytotype composition of populations was used as an explanatory variable and field-recorded environmental factors as dependent variables. The inset diagram shows correlations of field-recorded environmental factors with the first two canonical axes. Vectors of the categorical variables can be interpreted as the probability of their occurrence. Environmental variables with names in large font size are significantly correlated with at least one of the first two canonical axes (*P* < 0.001).

Both tests on the first (pseudo*F* = 1.2, *P* = 0.001) and all canonical axes (pseudo*F* = 1.9, *P* = 0.001) of constrained principal coordinate analysis revealed non-random differences in local-scale environmental conditions between groups of different cytotype compositions (Figure 5). However, all canonical axes collectively explained only 4.6% of the total variation among cytotype groups (PC1 = 2.9%, PC2 = 0.6%). The first canonical axis represented a gradient of synanthropy, with natural and semi-natural sites (rocky outcrop, grassland, dry, and mesic forests) with longer distances to the nearest arable land on the left to ruderalised or even synanthropic sites (*Robinia* forest, shrub, arable land & field margin) on the right of the ordination diagram. Except for triploid populations, single-ploidy populations were ordered along the first canonical axis from lower ploidy levels on the left to the higher ones on the right. Most mixed-cytotype groups of sites with the participation of pentaploids were usually placed in intermediate positions between uniform-ploidy sites of participating cytotypes. Other mixed-cytotype groups were usually placed near the uniform-cytotype sites of one of the participating cytotypes. The second canonical axis represents a gradient of increasing habitat heterogeneity. Cytotype groups placed in the upper part of the ordination diagram were usually mixed-cytotype sites in addition to uniform triploid populations. Paired tests between cytotypes revealed significant differences (after Bonferroni correction) in local-scale environmental conditions between lower (3*x*, 4*x*) and higher ploidy levels (6*x*, 7*x*), and between penta- and hexaploids (Supplementary Table 4).

## Discussion

### Cytotype Diversity

We found a great diversity of ploidy levels in *A. oleraceum*, ranging from triploids to octoploids. Our data on the distribution of lower ploidy levels (i.e., 2*n* = 3*x*−6*x*) correspond well to published chromosome counts, but significantly extends their distributional ranges (Figure 1). Moreover, two uncommon cytotypes corresponding to hepta- and octoploids are the first such counts within *A*. sect. *Codonoprasum* and represent extremely rare ploidy levels within *Allium* (Hanelt et al., 1992; Goldblatt and Johnson, 2010; Rice et al., 2015; Peruzzi et al., 2017), which is considered an example of a genus with extraordinary high intraspecific variation in ploidy levels (Han et al., 2020). Such substantial intraspecific ploidy variation is only rarely seen in plants, e.g., in *Cardamine yezoensis* Maxim. with six (Marhold et al., 2010) and *Oxalis obtusa* Jacq. with seven (Krejčíková et al., 2013) cytotypes, respectively.

The majority of analyzed plants and populations were pentaploid. Higher frequencies of odd-ploidy levels (especially >3*x*) are rare within ploidy-polymorphic species (Husband et al., 2013; Kolář et al., 2017) and odd-ploidy dominance has only exceptionally been documented in mixed-ploidy species (Mock et al., 2012; Šingliarová et al., 2019). High frequencies of odd-ploidy cytotypes might indicate frequent intercytotype hybridization between even-ploidy levels (Husband et al., 2013; Hanušová et al., 2019; Šingliarová et al., 2019), and odd cytotypes generate mostly aneuploids due to unequal meiotic division resulting in unbalanced chromosome segregation and chromosome elimination (Ramsey and Schemske, 1998, 2002). In contrast, odd-ploidy cytotypes of *A. oleraceum* (incl. pentaploids) formed cytotype-uniform populations more frequently than even-ploidy cytotypes (Table 2), and no aneuploids were found in natural populations despite karyological screening of more than 500 adult plants (Levan, 1933; Karpavičienė, 2007, 2012; Åström et al., 2015; this study). We suggest that the strong asexual mode of reproduction of *A. oleraceum* cytotypes (Fialová et al., 2014) and non-viability of aneuploid seeds are crucial factors behind the observed patterns. Indeed, other polyploid species with higher frequencies of odd-ploidy cytotypes exhibit almost exclusively asexual reproduction (e.g., Krahulcová and Jarolímová, 1993; Kao, 2007; Mock et al., 2012; Kolář et al., 2017).

### Drivers of Large-Scale Cytotype Distributions

The large-scale distribution pattern of different cytotypes throughout the species range was found to be remarkably complex. While tetra- and pentaploids were common across almost the entire species range and sympatric with other cytotypes at lower latitudes, other cytotypes occupied lower latitudes (below 52°) and were distributed parapatrically (tri- vs. hexaploids) or allopatrically (tri- vs. hepta- and octoploids; Figure 1). The current distribution of all cytotypes also overlaps the range of diploid, sexually reproducing hypothetical progenitors of the *A. paniculatum* complex (Stearn, 1980; Pastor and Valdés, 1983; Brullo et al., 1996a; Salmeri et al., 2016), but exclusively at lower latitudes. Considering both polyploid *A. oleraceum* and diploids of *A. paniculatum* complex, most regions with a high cytotype diversity are confined to unglaciated areas during the LGM while only polyploids are currently distributed at both higher latitudes and elevations, with tetra- and pentaploids being the only cytotypes represented in northern areas which were glaciated during the LGM (Huntley and Birks, 1983). A somewhat similar scenario has been described within several sexual–asexual taxa (e.g., *Antennaria* L., Bayer and Stebbins, 1987; *Ranunculus auricomus* group, Hörandl, 2006; *Crataegus* L., Lo et al., 2013; *Ranunculus kuepferi* Greuter & Burdet, Kirchheimer et al., 2016; Schinkel et al., 2016; *Paspalum intermedium* Munro ex Morong, Karunarathne et al., 2018), where polyploid apomicts tend to have larger ranges and occupy colder areas (i.e., higher latitudes and/or elevations) than diploid sexuals, suggesting evolutionary advantages of polyploidy associated with apomixis in the colonization of deglaciated areas (i.e., geographical parthenogenesis; Bierzychudek, 1985; Hörandl, 2009; Tilquin and Kokko, 2016; Hojsgaard and Hörandl, 2019). Below, we discuss the roles of several processes which might explain the current distribution patterns of *A. oleraceum* cytotypes, such as niche evolution, asexuality, and non-adaptive processes (e.g., biogeographic history, dispersal).

### Role of Niche Differentiation

There has been mixed support for the role of niche differentiation in polyploid establishment and evolution (Levin, 2002; Martin and Husband, 2009; Parisod et al., 2010; te Beest et al., 2012; Glennon et al., 2014; Visser and Molofsky, 2015; Marchant et al., 2016), although recent research using biogeographic data across vascular plants has proved that ecological niche differentiation is an important component of polyploid speciation and that polyploids have a faster niche differentiation than their diploid relatives (Baniaga et al., 2020). Unfortunately, our study does not directly compare niches between polyploids and diploid progenitors to test the phylogenetic niche conservatism (Wiens et al., 2010), since the diploid progenitors of *A. oleraceum* are not known with certainty (Duchoslav et al., 2010). Putative diploid ancestors of *A. oleraceum* (e.g., *A. paniculatum, A. podolicum* Blocki ex Racib. & Szafer, and *A. fuscum* Waldst. & Kit.; Levan, 1937) however share two characteristics: (i) their niches are restricted to a narrow range of natural habitats (rocky outcrops and dry grasslands) in (ii) dry and warm regions of southeastern Europe (Stearn, 1980; Brullo et al., 1996a, 2001; Dobrotchaeva et al., 1999; Ciocârlan, 2000; Ghendov, 2015; Salmeri et al., 2016). In contrast, the niche of polyploid *A. oleraceum* also comprises more mesic (colder and moister) climatic conditions (Figures 1–3) and a wide spectrum of habitat types, including fertile weedy habitats (Figure 4). The stronger synanthropic affinity of polyploids, in contrast to their diploid congeners, was recently found in several polyploid complexes (Zozomová-Lihová et al., 2014; Chung et al., 2015; Němečková et al., 2019; Rejlová et al., 2019). Exact niche comparison between polyploid *A. oleraceum* and its diploid progenitors is needed, as soon as molecular analyses reveal the diploid progenitors of *A. oleraceum*.

Using coarse-grained environmental variables, we identified high niche overlap in most pairwise comparisons between *A. oleraceum* cytotypes (Table 3, Figure 2). Even though the niche equivalency and similarity tests do not allow us to confirm significant differentiation or similarity in most cases, we identified two patterns of niche change within this polyploid series. Whereas, we found evidence of both niche expansion and innovation in tetraploids compared to triploids, higher ploidy levels showed (almost) zero niche expansion, but a trend of increasing unfilling of tetraploid niche. Niche unfilling in higher ploidy levels was primarily caused by the contraction of niche envelopes toward a lower continentality of the climate and resulted in a gradual decrease of niche breadth and a gradual shift in niche optima in higher (>4*x*) ploidy levels. Consequently, the geographic ranges of most cytotypes overlapped to various extents, with wider ranges of tetra- and pentaploids due to their wider niche breadth. Also analysis of local-scale environmental variables demonstrated that higher ploidy levels (6*x*, 7*x*) tend to occupy a narrower segment of habitats occupied by lower ploidy levels (4*x*, 5*x*), but without any sign of niche novelty. In agreement with some recent studies in other polyploid complexes (e.g., Brittingham et al., 2018; Gaynor et al., 2018; Hanušová et al., 2019), the observed divergent patterns of niche changes among *A. oleraceum* cytotypes seem to contradict the classical adaptive evolutionary scenario (Levin, 1975, 2002; Fowler and Levin, 1984, 2016), assuming shift in ecological niches of higher polyploids (i.e., niche innovation or niche expansion) compared to their lower ploidy level relatives.

One likely explanation of the observed niche patterns in *A. oleraceum* polyploids is linked to the type of polyploidy because autopolyploidy, in contrast to allopolyploidy, does not inevitably produce transgressive traits to fuel adaptive ecological divergence (Hegarty and Hiscock, 2008; Parisod et al., 2010). Additionally, multiple origins of polyploids (Soltis et al., 2010) might affect the genetic variation and consequently niche breadth of polyploids as was recently shown in e.g., tetraploids of *Dianthus broteri* Boiss. & Reut. (López-Jurado et al., 2019) and *Jasione maritima* (Duby) Merino (Castro et al., 2020). Our previous study of genome size variation in *A. oleraceum* cytotypes revealed a remarkably complex pattern both within and between cytotypes (Duchoslav et al., 2013). Specifically, 4*x*−7*x* cytotypes from (south-)western Europe had similarly sized monoploid genome sizes (1Cx), which might suggest their (recent) autopolyploid origin (Balao et al., 2009), whereas differently sized 1Cx of the eastern 3*x*−5*x* cytotypes could be the result of independent polyploidization events, potentially involving crosses between different diploids of the *A. paniculatum* complex (Duchoslav et al., 2013). Moreover, populations of two cytotypes (4*x*, 5*x*), whose distributions cover both western and eastern parts of Europe (Figure 1), also diverged in genome sizes in a west-east direction (Duchoslav et al., 2013; Supplementary Figure 2). Consequently, we suppose that both tetra- and pentaploids are assemblages of different evolutionary lineages arising from different environmental conditions (Duchoslav et al., 2013), i.e., their niches are actually a composition of narrower niches of lineages with different origins. If the lineages are considered as one unit (= cytotype), they show a large niche breadth whereas other cytotypes distributed in either western (6*x*, 7*x*) or eastern (3*x*) parts of the species range just show a subset of broad niches of tetra- and pentaploids (Figures 2, 3). Two additional pieces of evidence support outlined explanations. Firstly, common garden experiments and physiological measurements on Central European populations of tetra-, penta-, and hexaploids have generally shown similar competitive ability (Fialová and Duchoslav, 2014) and photosynthetic capacity (Ježilová et al., 2015) of cytotypes, but also significantly lower interpopulation variation of the studied parameters in hexaploids than in tetra- and pentaploids. Secondly, a study of allozymes revealed more genetically differentiated populations of Central European tetra- and pentaploids, with two-fold higher total and within-population diversity, in comparison with genetically uniform hexaploids (Duchoslav and Staňková, 2015).

Triploids had the narrowest niche of all cytotypes with a niche optimum resembling the ecological characteristics of putative diploid progenitors (Čeřovský et al., 1999; Ciocârlan, 2000; Ghendov, 2015; Salmeri et al., 2016). Moreover, triploids only slightly exceed the geographic range of putative diploid progenitors. The narrow niche of triploids may be explained by their complete sterility (Jírová, 2007), a factor that hinders the activation of natural selection (see below; Chung et al., 2015).

The narrower range sizes and niches of higher ploidy levels (6*x*, 7*x*) could also be explained by their more recent origin. Despite several competing hypotheses as to how species (cytotype) “age” may affect range size (Johnson et al., 2014; Brittingham et al., 2018; Sheth et al., 2020), and the unknown evolutionary age of *A. oleraceum* cytotypes, newly arising higher ploidy levels (6*x*, 7*x*) certainly face competition from already established lower ploidy levels (4*x*, 5*x*). Niche contraction in higher ploidy levels has previously been observed in several polyploid complexes (Theodoridis et al., 2013; Gaynor et al., 2018). Furthermore, niche-contraction in higher ploidy levels to fertile sites rich in nitrogen and phosphorus (i.e., field margins and road ditches, *Robinia pseudoacacia* forests) might be affected by their larger genome sizes (Duchoslav et al., 2013). Both cytotypes have very large genomes (*sensu* Leitch et al., 1998), which has been suggested to have increased expenses of building and maintaining nucleic acids and associated proteins which may, under certain circumstances, act as selection pressure (Šmarda et al., 2013; Guignard et al., 2016, 2017). Indeed, the concentration of available phosphorus was found to be significantly higher in soils of Central-European sites of hexaploids than in soils of sites of tetra- and pentaploids (Duchoslav et al., 2010). Additionally, plants of higher ploidy levels with large genomes might also be constrained by the threshold for minimum stomatal size and therefore be less tolerant of water stress due to a larger stomatal length (Veselý et al., 2012). Consequently, their spread might be constrained in more arid climates.

### Asexuality

Asexuality has long been hypothesized as a viable mechanism (Gustafsson, 1948; Yamauchi et al., 2004; Husband et al., 2013; Herben et al., 2017) by which newly emerging or immigrating polyploids might establish at a local site. It enables them to persist in face of strongly reduced fitness either due to mating with their more common progenitors or relatives of other ploidy levels (minority cytotype exclusion; Levin, 1975) or to meiotic irregularities caused by the formation of various meiotic configurations causing a reduction of the seed set (Ramsey and Schemske, 2002). Asexuality may also play a decisive role in the colonization of new areas, e.g., during the recolonization of previously glaciated habitats (Hörandl, 2009). Due to the ability to reproduce without mates or pollinators, such as asexually formed seeds in apomicts (Hörandl, 2006), a new population can establish from a single propagule (“Baker's Law,” Baker, 1967). However, an application of this scenario on polyploid *A. oleraceum* plants is puzzling. Even though *A. oleraceum* is not an apomictic plant (*sensu* Asker and Jerling, 1992), its cytotypes predominantly reproduce uniparentally by huge and similar numbers of asexual propagules in the form of bulbils formed in the inflorescence (Fialová et al., 2014). Bulbils are ecologically similar to seeds (Ronsheim, 1994), but have a higher amount of stored resources (Karpavičienė and Karanauskaitė, 2010) and higher germination rates (Fialová et al., 2014), expressed in a higher survival and growth rate of plants originating from bulbils in comparison with seedlings (Fialová and Duchoslav, 2014). Consequently, bulbils increase the probability of successful establishment of a new population in various types of habitats differing in intensity of competition (e.g., open early-successional vegetation, dense grasslands, shaded forests; Fialová and Duchoslav, 2014; Duchoslav et al., 2017). The abovementioned traits favor *A. oleraceum* cytotypes over their putative sexual diploid progenitors lacking the ability of aerial bulbil formation (Stearn, 1980; Salmeri et al., 2016), but cannot favor one cytotype over others. However, two cytotypes with the widest niche breadth (tetra- and pentaploids) produce an order of magnitude more sexual seeds than other cytotypes (Karpavičienė, 2012; Fialová et al., 2014). Such a combination of sexual and asexual reproduction potentially advantages tetra- and pentaploids over other cytotypes due to their better ability to adapt, together with the conservation of such genetically and ecologically different genotypes via asexual propagules (= “Frozen Niche Variation Model”; Vrijenhoek, 1994). In contrast, cytotypes with scarce sexual reproduction may have a restricted niche because local adaptation by recombination will occur at lower rates, as recently shown in apomictic tetraploids of *Ranunculus kuepferi* (Kirchheimer et al., 2016).

### Historical Factors

Despite high niche overlap between cytotypes and spatial intermingling of most cytotypes at a landscape scale, we detected some often quite large, nearly or entirely ploidy-uniform areas in different parts of the species' range (Figure 1). Most of them were formed by either penta- or tetraploids, although these two cytotypes have identical niches. One possible explanation could be that there are other environmental factors not explored here which may be responsible for niche differentiation between cytotypes (e.g., Zozomová-Lihová et al., 2014). However, we rather suggest that movements of cytotypes during and following post-glacial migration may have resulted in founder effects (te Beest et al., 2012; McAllister et al., 2015). Some of such founder effects might be of anthropogenic origin owing, for example, to the strong connection of *A. oleraceum* to Iron Age habitation and ancient settlements in the Nordic countries (Hæggström and Åström, 2005). For example, the dominance of pentaploids in southwestern Finland but prevalence of tetraploids in southeastern Finland was associated with sailing routes from Sweden dominated by pentaploids and old trade routes of pedlars from Russia dominated by tetraploids (Åström et al., 2015). Other ploidy-uniform areas, dominated by hexaploids, were found in e.g., the lowlands of the Friuli-Venezia Giulia and Veneto regions in northern Italy and the hills of southern Bohemia in the Czech Republic (Figure 1). Both regions are, however, strongly man-influenced landscapes with low habitat heterogeneity, dominated by intensively cultivated arable fields. We suggest that the interaction between affinity of hexaploids to fertile ruderal habitats and founder effect might be a viable explanation.

In addition, there is one large ploidy-uniform region dominated by pentaploids with rare occurrence of 3*x* + 5*x* mixed populations, ranging from the northwestern part of Hungary to northeastern Croatia and central Serbia. This region (Pannonian basin) has a subcontinental climate and large areas covered by loess, with degraded forest-steppe landscapes, surrounded by mountain ranges (Zólyomi and Fekete, 1994). Currently, we can only speculate about the causes of the observed pattern but we hypothesize that these pentaploids represent a lineage adapted to these specific environmental conditions.

The surprising absence of triploids from other southern regions of Europe (Iberian Peninsula, France, Italy) is hardly explainable as a sampling bias (we sampled more intensively in these regions than in Eastern Europe). More likely, their absence or perhaps rareness coincides with the apparently low diversity of putative diploid progenitors in the western part of the Mediterranean (Spain, France) than in the eastern part (e.g., Brullo et al., 1997; Jauzein and Tison, 2001; Bogdanović et al., 2008; Aedo, 2013; Tison and de Foucault, 2014; Brullo and Guarino, 2017), which may decrease the probability of triploid formation by fusion of reduced and unreduced gametes in crosses between diploid progenitors or by crosses between diploid progenitors and tetraploids of *A. oleraceum*. The latter explanation is supported by newly discovered high-level polyploids (hepta- and octoploids) in *A. oleraceum* found in southwestern Europe. Such high ploidy levels could have originated via various pathways including auto- and/or allopolyploidy (see also below), with participation of polyploid species of the *A. paniculatum* complex (e.g., tetraploid *A. oporinanthum* Brullo, Pavone & Salmeri), which are reported from the northwestern Mediterranean (Brullo et al., 1997).

A sharp boundary of the continuous distribution of hexaploids was observed in the Western Carpathians, where hexaploids meet triploids of eastern origin (Figure 1). This contact zone could be considered as secondary, with hexaploids migrating from the west. Concordant patterns of cytotype contact zones have been detected in several different polyploid complexes in this region (Mráz and Ronikier, 2016). However, the discovery of rare hexaploids in the Transylvanian basin, separated from the easternmost localities of the continuous range by a c. 300 km disjunction, could be explained as the result of independent origin of this cytotype (see also below).

### Mixed-Ploidy Populations: Their Origin and Persistence

A high frequency of mixed-ploidy populations has been recently observed in several plant species (Kolář et al., 2017 and references therein; Hanušová et al., 2019) despite theoretical arguments that the coexistence of multiple cytotypes within populations may be unstable and represent only a transient stage following frequent generation or immigration of a divergent cytotype (Levin, 1975). We found 17.4% of populations comprising two or three cytotypes within *A. oleraceum*, which is within the mean estimates at the population level for mostly sexual and mostly asexual mixed-ploidy species as reviewed by Kolář et al. (2017). However, the frequency of mixed-ploidy populations in *A. oleraceum* observed in our study likely undervalues its real estimate because of a positive correlation between sampling effort (area, number of individuals) and within-population cytotype richness (see also Sonnleitner et al., 2010; Šingliarová et al., 2019).

We revealed mixed-ploidy populations across most of the studied species range, confirmed all previously reported combinations of *A. oleraceum* cytotypes composing mixed-ploidy populations (Duchoslav et al., 2010), and discovered five new cytotype combinations (Table 2). We regard the origin and spatial distribution of the majority of these mixtures to be most likely explained by secondary contacts between cytotypes (*sensu* Petit et al., 1999). The following arguments support the “secondary contact hypothesis” in *A. oleraceum*. Firstly, most mixed-ploidy populations occur exclusively in contact zones of participating cytotypes, in a diffuse, mosaic-like pattern with uniform-cytotype populations, not as rare cases of cytotype mixtures in otherwise ploidy-uniform areas. Furthermore, all cytotypes also form their own populations, suggesting a rather long term stability of all cytotypes and not a scenario of ongoing polyploidisation via the production of unreduced gametes, as proposed, for example, for hexaploids and higher ploidy levels of *Cardamine yezoensis* (Marhold et al., 2010), or a scenario of repeated interploidy hybridization such as in the case of the assumed origin of pentaploids in mixed 4*x* + 6*x* populations of *Cystopteris fragilis* (L.) Bernh. (Hanušová et al., 2019). In these examples, newly generated polyploids do not spread from mixed populations and do not develop their own populations. Secondly, the origin of some mixed-ploidy populations in *A. oleraceum* (e.g., 4*x* + 5*x*, 4*x* + 7*x*; 5*x* + 7*x*) is difficult to explain as a result of interploidy crosses or of primary formation of a higher cytotype in lower-ploidy level populations (Ramsey and Schemske, 1998, 2002). Thirdly, the observed moderate to strong overlaps of both niches and geographic distributions between most cytotypes may result in local spatial contacts, which are significantly enhanced by the dispersion of propagules (especially aerial bulbils) via human-mediated transport of hay and cereals, soil movement during soil preparation, or collection and spreading of bulbils by small rodents (Duchoslav, 2001). Fourthly, short-time cytotype coexistence in *A. oleraceum* was experimentally evidenced in the form of survival of foreign ploidy levels at home-ploidy sites in a 5-year experiment with aerial bulbils transplanted reciprocally to cytotype-uniform sites of tetra-, penta-, and hexaploids in central Europe (Duchoslav et al., 2017). Lastly, asexual reproduction as a dominant reproductive mode of all cytotypes (Fialová et al., 2014) facilitates their establishment (Fialová and Duchoslav, 2014) and allows established plants to avoid reproduction costs due to their minority status (Yamauchi et al., 2004; McAllister et al., 2015) and potential lack of compatible partners for mating in this allogamous species. The decisive role of asexuality for local coexistence of different cytotypes was proved by Kao (2007, 2008), who observed no reduction in fitness of rare cytotypes in mixed-cytotype populations in *Arnica cordifolia* Hook, a facultatively asexual species.

Nevertheless, we cannot rule out that some mixed populations represent the primary zone of contacts between cytotypes or that gene flow between cytotypes occurs. An allozyme study of Czech populations of *A. oleraceum* (Duchoslav and Staňková, 2015) demonstrated complex patterns of genetic similarity between tetra-, penta-, and hexaploid plants in mixed-ploidy populations, ranging from close to weak relatedness between locally coexisting cytotypes. The identical banding pattern of tetra- and hexaploids observed in two 4*x* + 6*x* populations was interpreted as hexaploids having originated *in-situ de novo* via fusion of unreduced and reduced gametes of tetraploids (Duchoslav and Staňková, 2015). However, we believe that a neohexaploid origin in tetraploid populations should be a rare process given the fact that no hexaploid plants were found in tetraploid populations outside the continuous range of hexaploids (Figure 1). On the other hand, the discovery of rare octoploids in a tetraploid population observed in central Italy (No. 452; Figure 1) might be interpreted as a spontaneous polyploidization event.

Higher odd-ploidy cytotypes (especially pentaploids) have a relatively higher fitness than triploids due to an increased production of viable euploid gametes ranging from 1*x* to 5*x* (Ramsey and Schemske, 1998; Costa et al., 2014). These may thus participate in both intra- and interploidal crosses (Sutherland and Galloway, 2017). However, no evidence of the occurrence of adult plants of other-than-mother cytotypes was observed in populations in ploidy-uniform regions (Figure 1), even though some of those regions were dominated by pentaploids, potentially producing karyologically variable progeny. Cytotype uniformity within such populations could be explained by a weak competitive ability and consequently lower survival of seedlings in comparison with young plants sprouted from bulbils, i.e., asexual copies of the maternal cytotype (Fialová and Duchoslav, 2014). Further studies into the cytotype variation of generative offspring produced by each cytotype and inter-cytotype crossing experiments are needed to evaluate the role of gene flow between cytotypes as a potential source of genetic and ecological diversity in *A. oleraceum*.

## Conclusions

Our study has provided detailed insight into the diversity and ploidy distribution patterns in populations of a geophytic species at a continental scale. Large ploidy diversity (six ploidy levels; 3*x*−8*x*) ranks *A. oleraceum* among the most cytotype-diverse mixed-ploidy plant species (Kolář et al., 2017), and the dominance of pentaploids (50.2%) makes *A. oleraceum* a unique case. All types of spatial arrangements of cytotypes at the landscape scale (i.e., allopatry, parapatry, and sympatry) were detected in *A. oleraceum*, together with relatively frequent coexistence of multiple ploidy levels within populations, resulting in various cytotype combinations. The widespread distribution of tetraploids and pentaploids contrasted with narrower ranges of both lower (triploid) and higher ploidy levels (hexa- to octoploids), which also differed from each other in geographic distributions.

We further focused on possible evolutionary drivers of the observed spatial patterns of cytotypes. In contrast to the classical adaptive evolutionary scenario, assuming niche innovation or niche expansion in higher ploidy levels, we found niche expansion in tetraploids compared to triploids followed by gradual niche contraction in higher ploidy levels, with higher ploidy levels occupying mainly synanthropic habitats. The wide niche breadth of tetraploids and pentaploids might be explained by their multiple origins from different environmental conditions, higher “age,” and retained sexuality, which probably preserves their adaptive potential. By contrast, both lower and higher ploidy levels with narrower niches are mostly asexual and probably originated in a limited range of contrasting environments. Newly evolved higher ploidy levels have also faced competition from existing cytotypes, which might have affected a shift of their niche. Persistence of local ploidy mixtures could be enabled by the perenniality of *A. oleraceum*, and intensive vegetative reproduction of all cytotypes, which facilitates establishment and allows plants to avoid reproductive costs due to their minority status. Vegetative reproduction might also significantly accelerate colonization of new areas, including recolonization of previously glaciated habitats. Further study should primarily focus on molecular analyses to reveal the relationships between cytotypes and to identify their origin. Besides that, comparison of the niches of polyploids with those of identified diploid progenitors could also be tested.

## Data Availability Statement

The original contributions presented in the study are included in the article/Supplementary Materials, further inquiries can be directed to the corresponding author/s.

## Author Contributions

MD designed the research and conducted field collection with the participation of MJ, LŠ, and KV. MJ, LK, and LŠ conducted laboratory analyses. JB extracted geoinformatics data and draw maps. MD performed statistical analyses and wrote the manuscript with help of JB and LK. All authors participated in the discussion and approved the submitted version.

## Conflict of Interest

The authors declare that the research was conducted in the absence of any commercial or financial relationships that could be construed as a potential conflict of interest.

## Supplementary Material

Supplementary Figure 1 Microphotographs of mitotic plates of *Allium oleraceum* cytotypes (2*n* = 3*x* − 8*x*). Scale bar = 10 μm. Plant locations: 3*x*: Ukraine (Population No. 234), 4*x*: Czech Republic (No. 1118), 5*x*: Czech Republic (No. 1091), 6*x*: Czech Republic (No. 1147), 7*x*: France (No. 80), 8*x*: Spain (No. 239).

Supplementary Figure 2 **(A)** Box-plots of mean relative fluorescence (ratio to the internal standard *Triticum aestivum* cv. *Saxana*) of 596 populations of six ploidy levels of *Allium oleraceum* measured by FCM (PI stain; see Table 1). **(B, C)** Geographic pattern of the mean relative fluorescence of *Allium oleraceum* cytotypes along latitude **(B)** and longitude **(C)**, respectively. The median smoothed line was fitted separately for each cytotype by moving average procedure where running medians are used instead of running means. Chromosome counts were done for selected populations of tetra- and pentaploids with marginal relative fluorescence. Such populations are marked by asterisks (*) in the plots B and C.

Supplementary Figure 3 Detailed distribution of ploidy levels of *Allium oleraceum* over its range, based on all available data (Supplementary Table 1). Separate maps were drawn for each dominant cytotype including ploidy-mixed populations. Different ploidy levels are distinguished by different colours: 3*x* - yellow, 4*x* - green, 5*x* - blue, 6*x* - red, 7*x* - purple, 8x - orange. Ploidy-mixed populations are depicted as a pie chart showing the local frequency of cytotypes. The size of the circle represents the number of analysed individuals per population. The lines with corresponding hatching indicate the limits of the continuous permafrost (light brown line) and glaciation (light blue line or polygons) during the last permafrost maximum according to Lindgren et al. (2016). The inset map shows the distribution of cytotypes in northern Norway. The black line shows the northern range limit of diploids of *Allium paniculatum* complex, compiled from various sources (see Material and Methods).

Supplementary Figure 4 Detailed distribution of ploidy levels of *Allium oleraceum* in Central Europe, based on all available data (Supplementary Table 1). Different ploidy levels are distinguished by different colours: 3*x* - yellow, 4*x* - green, 5*x* - blue, 6*x* - red, 7*x* - purple, 8x - orange. Ploidy-mixed populations are depicted as a pie chart showing the local frequency of cytotypes. The size of the circle represents the number of analysed individuals per population. The lines with corresponding hatching indicate the limits of the continuous permafrost (light brown line) and glaciation (light blue line or polygons) during the last permafrost maximum according to Lindgren et al. (2016). The inset map shows the distribution of cytotypes in northern Norway. The black line shows the northern range limit of diploids of *Allium paniculatum* complex, compiled from various sources (see Material and Methods).

Supplementary Figure 5 Frequency distribution (%) of categories of the following field-recorded, fine-scale environmental variables at sites of different cytotypes of *Allium oleraceum*: habitat heterogeneity (number of local habitats occupied by the local population: white = 1 habitat, grey = 2 habitats, black = 3 habitats); Light conditions (white = deep shade, light grey = half shade, dark grey = light shade, black = full insolation). The bottom panels represent percentile-plots of elevation and heat load at sites of different cytotypes of *A. oleraceum*.

Supplementary Table 1 *Allium oleraceum* populations analysed in this study, including extracted data from other sources. For each population, ID code, locality description, geographic coordinates (WGS-84), sample size (Nind = number of individuals analysed; in the case of literature sources without sample size information, just one analysed individual per ploidy was considered for each site), ploidy level (Ploidy composition, ‘+’ means the case of mixed-cytotype populations), counted chromosome numbers, mean, SD, and sample size (Nindi) of the relative fluorescence intensity of identified cytotypes (flow-cytometry (FCM); *Triticum aestivum* cv. *Saxana* as an internal standard); dye used for FCM estimation (PI, DAPI), field-recorded, local-scale ecological variables and extracted coarse-grained environmental variables (see Supplementary Table 3 for explanation), and sources of data are given. Populations, which were used for ecological analyses, are marked with an asterisk within the column named “Niche analyses”.

Supplementary Table 2 Survey of coarse-grained environmental variables used in ecological niche analysis (PCAenv). For each variable, its abbreviation, units, scaling factor, and source of data are presented. Additionally, descriptive statistics (mean and SD) of each coarse-grained environmental variable for each cytotype, using all localities in Supplementary Table 1, are presented.

Supplementary Table 3 Survey of local-scale environmental and population variables used in the study. Vegetation types according to Mucina et al. (2016), which were included within each habitat type used in the study, are given at the bottom.

Supplementary Table 4 Summary of the pairwise multivariate analyses between cytotypes using db-RDA applied to local-scale environmental variables recorded in populations of *A. oleraceum*. The significance level of the db-RDA was assessed by Monte Carlo permutation test using 999 permutations. *P*-values in bold are significant after Bonferroni correction (*P* < 0.006).
