# Supplementary figures and images for: Intricate Distribution Patterns of Six Cytotypes of Allium oleraceum at a Continental Scale: Niche Expansion and Innovation Followed by Niche Contraction With Increasing Ploidy Level
Source: Front Plant Sci. 2020 Dec 9;11:591137. doi: 10.3389/fpls.2020.591137 (PMC7755979; doi:10.3389/fpls.2020.591137)

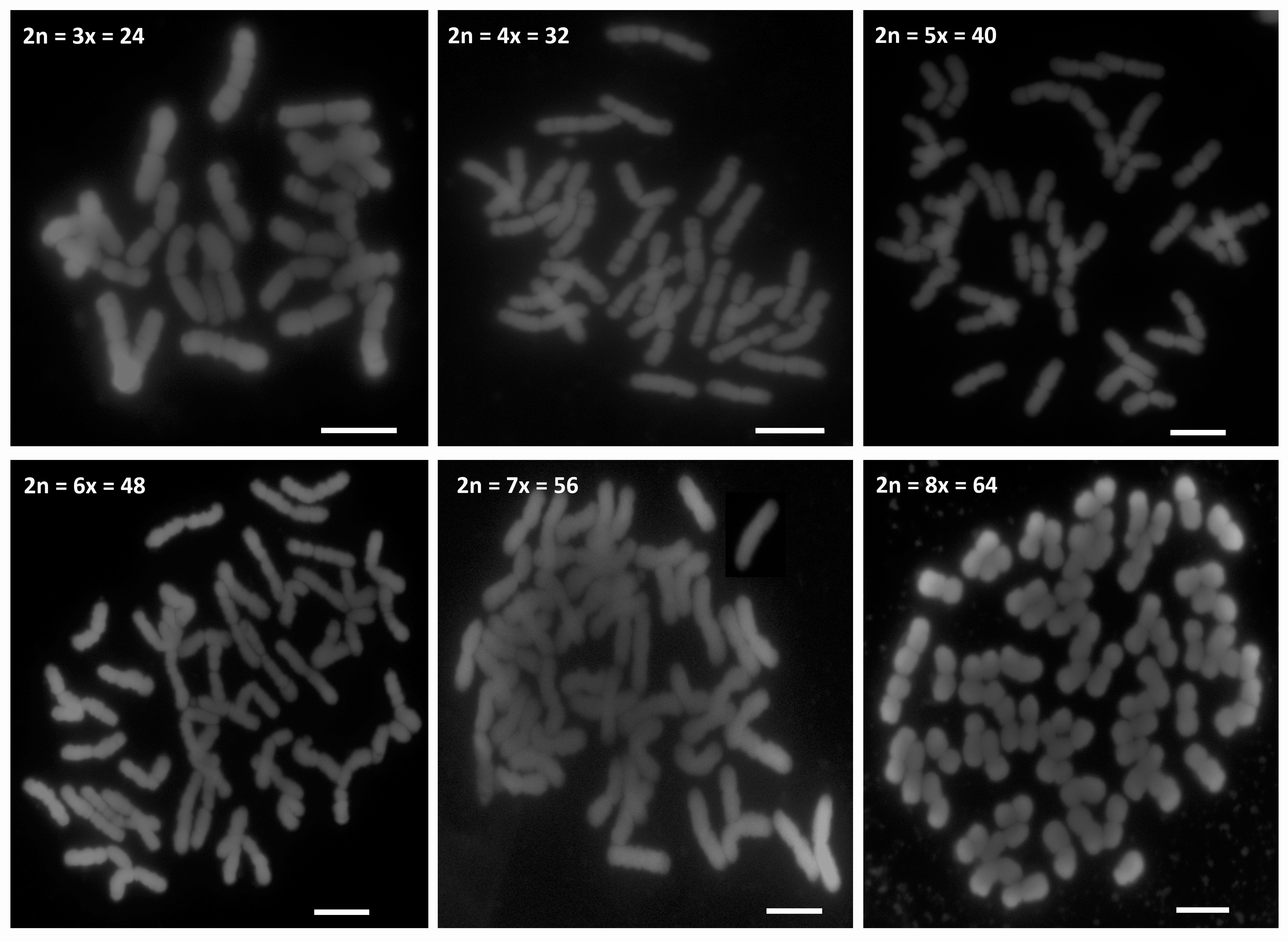

Supplement: Supplementary Figure 1 — Microphotographs of mitotic plates of Allium oleraceum cytotypes (2n = 3x − 8x). Scale bar = 10 μm. Plant locations: 3x: Ukraine (Population No. 234), 4x: Czech Republic (No. 1118), 5x: Czech Republic (No. 1091), 6x: Czech Republic (No. 1147), 7x: France (No. 80), 8x: Spain (No. 239). [file Image_1.jpg]

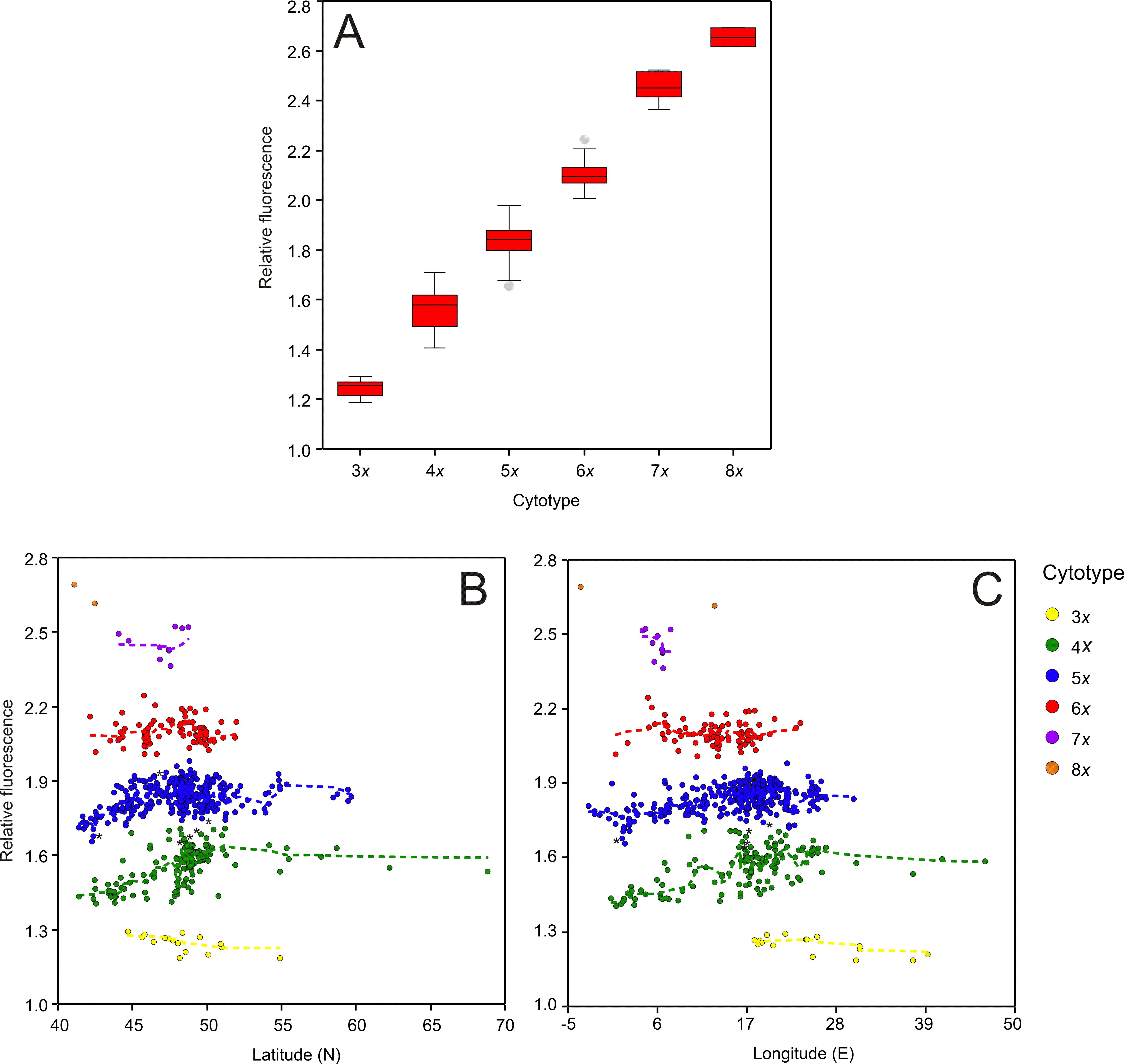

Supplement: Supplementary Figure 2 — (A) Box-plots of mean relative fluorescence (ratio to the internal standard Triticum aestivum cv. Saxana) of 596 populations of six ploidy levels of Allium oleraceum measured by FCM (PI stain; see Table 1). (B, C) Geographic pattern of the mean relative fluorescence of Allium oleraceum cytotypes along latitude (B) and longitude (C), respectively. The median smoothed line was fitted separately for each cytotype by moving average procedure where running medians are used instead of running means. Chromosome counts were done for selected populations of tetra- and pentaploids with marginal relative fluorescence. Such populations are marked by asterisks (*) in the plots B and C. [file Image_2.jpg]

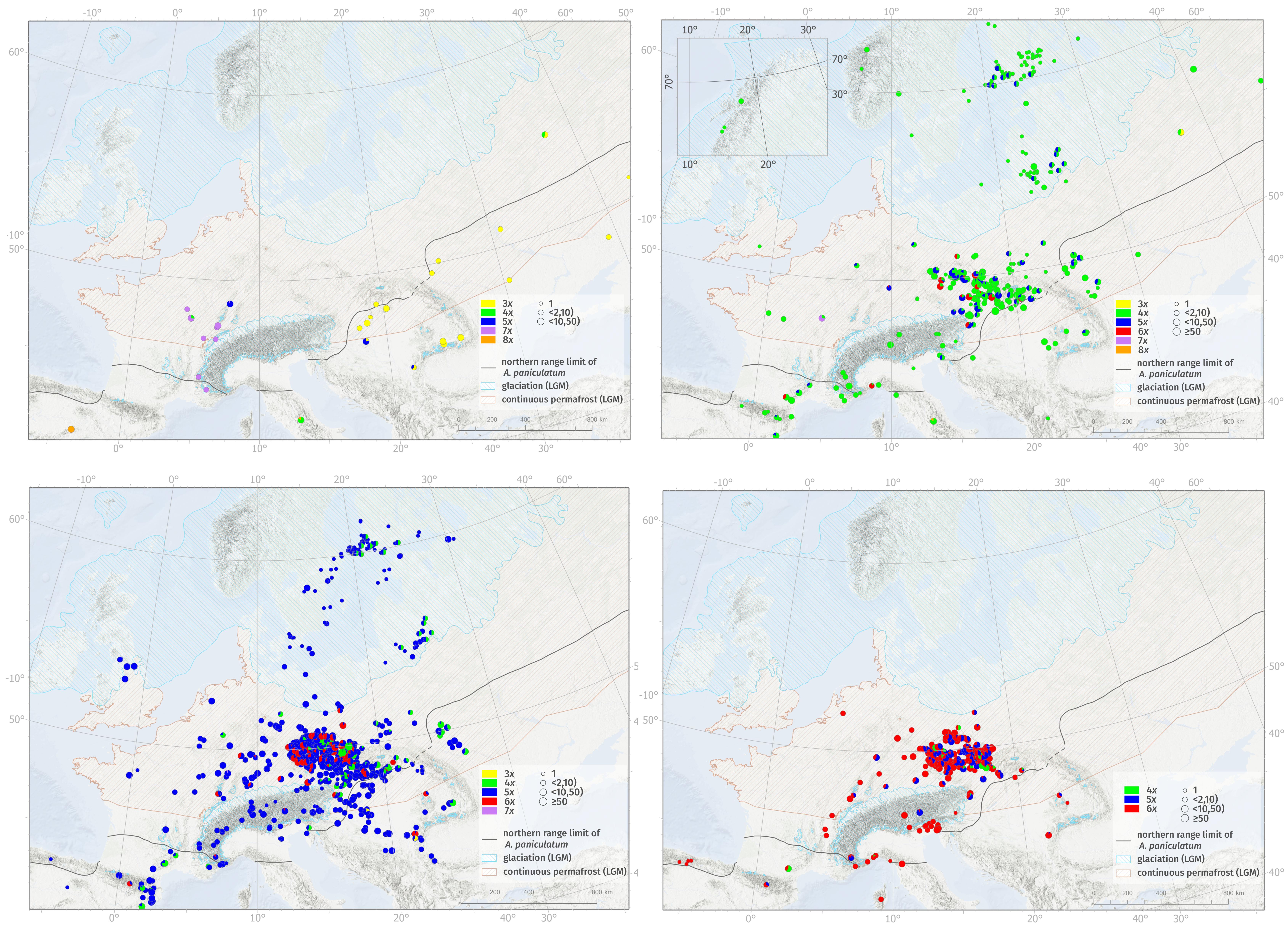

Supplement: Supplementary Figure 3 — Detailed distribution of ploidy levels of Allium oleraceum over its range, based on all available data (Supplementary Table 1). Separate maps were drawn for each dominant cytotype including ploidy-mixed populations. Different ploidy levels are distinguished by different colours: 3x - yellow, 4x - green, 5x - blue, 6x - red, 7x - purple, 8x - orange. Ploidy-mixed populations are depicted as a pie chart showing the local frequency of cytotypes. The size of the circle represents the number of analysed individuals per population. The lines with corresponding hatching indicate the limits of the continuous permafrost (light brown line) and glaciation (light blue line or polygons) during the last permafrost maximum according to Lindgren et al. (2016). The inset map shows the distribution of cytotypes in northern Norway. The black line shows the northern range limit of diploids of Allium paniculatum complex, compiled from various sources (see Material and Methods). [file Image_3.jpg]

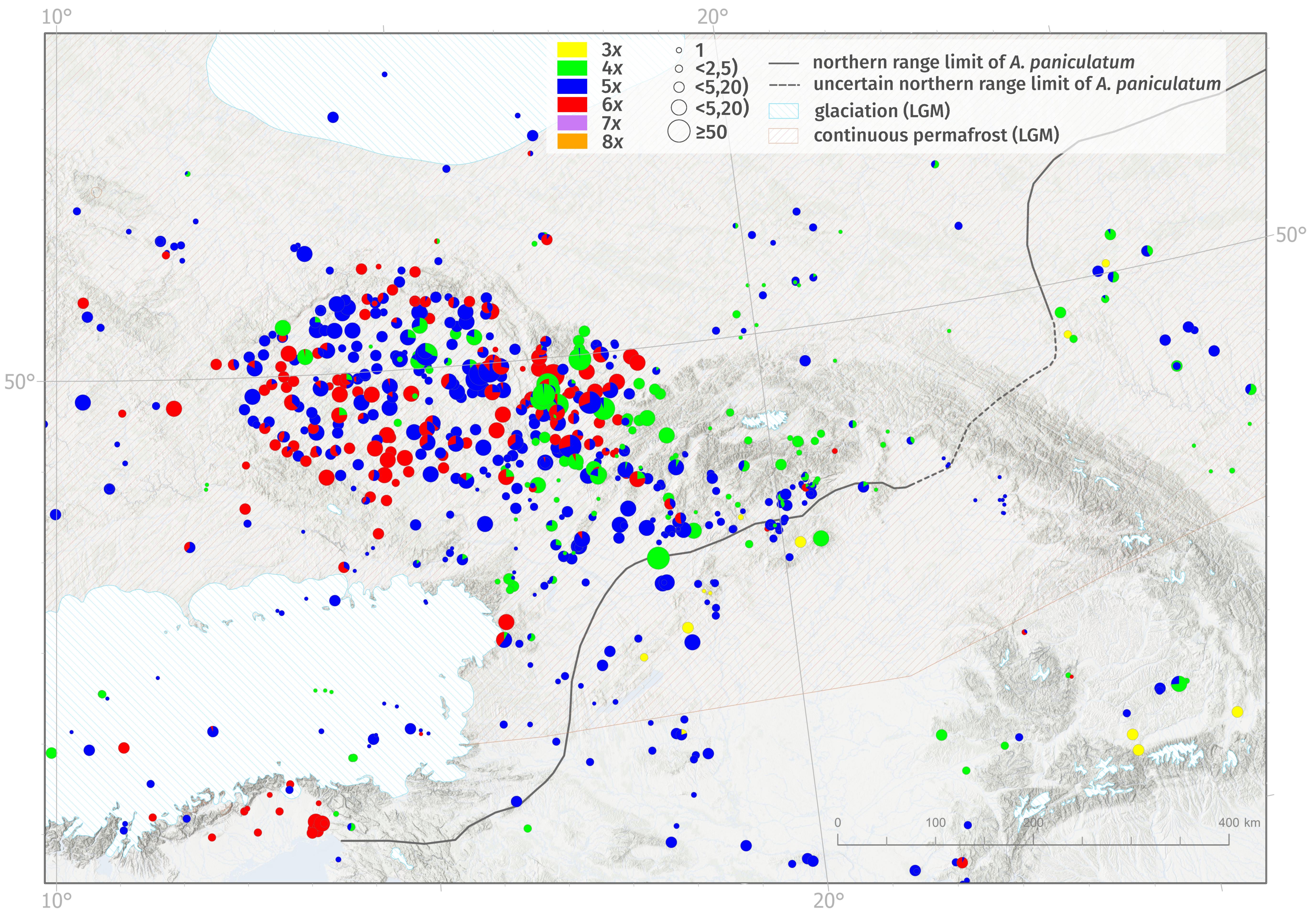

Supplement: Supplementary Figure 4 — Detailed distribution of ploidy levels of Allium oleraceum in Central Europe, based on all available data (Supplementary Table 1). Different ploidy levels are distinguished by different colours: 3x - yellow, 4x - green, 5x - blue, 6x - red, 7x - purple, 8x - orange. Ploidy-mixed populations are depicted as a pie chart showing the local frequency of cytotypes. The size of the circle represents the number of analysed individuals per population. The lines with corresponding hatching indicate the limits of the continuous permafrost (light brown line) and glaciation (light blue line or polygons) during the last permafrost maximum according to Lindgren et al. (2016). The inset map shows the distribution of cytotypes in northern Norway. The black line shows the northern range limit of diploids of Allium paniculatum complex, compiled from various sources (see Material and Methods). [file Image_4.jpg]

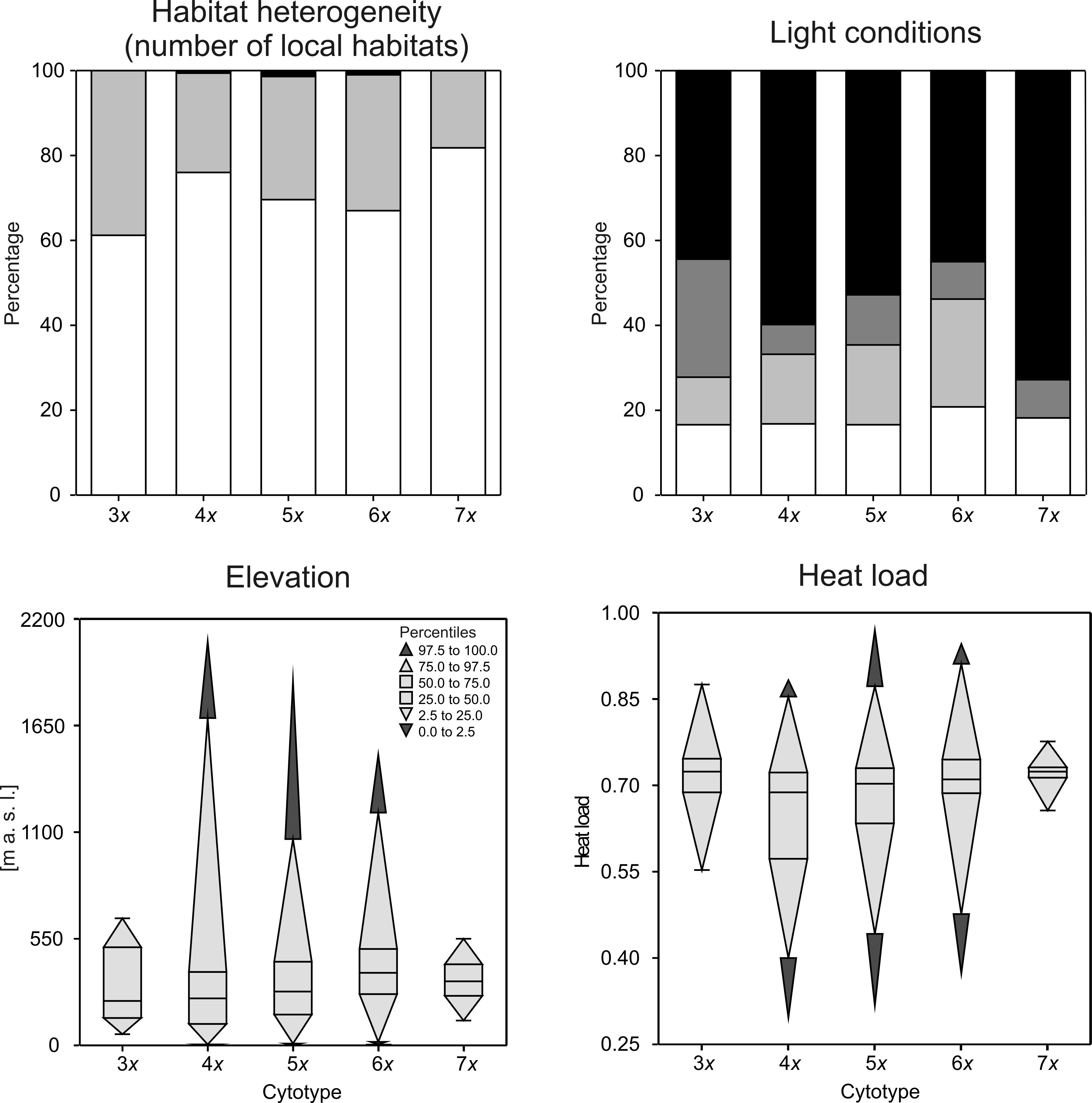

Supplement: Supplementary Figure 5 — Frequency distribution (%) of categories of the following field-recorded, fine-scale environmental variables at sites of different cytotypes of Allium oleraceum: habitat heterogeneity (number of local habitats occupied by the local population: white = 1 habitat, grey = 2 habitats, black = 3 habitats); Light conditions (white = deep shade, light grey = half shade, dark grey = light shade, black = full insolation). The bottom panels represent percentile-plots of elevation and heat load at sites of different cytotypes of A. oleraceum. [file Image_5.jpg]
